# Eukaryotic initiation factor 4A2 promotes experimental metastasis and oxaliplatin resistance in colorectal cancer

**DOI:** 10.1186/s13046-019-1178-z

**Published:** 2019-05-14

**Authors:** Zhan-Hong Chen, Jing-Jing Qi, Qi-Nian Wu, Jia-Huan Lu, Ze-Xian Liu, Yun Wang, Pei-Shan Hu, Ting Li, Jin-Fei Lin, Xiang-Yuan Wu, Lei Miao, Zhao-Lei Zeng, Dan Xie, Huai-Qiang Ju, Rui-Hua Xu, Feng Wang

**Affiliations:** 1 Department of Medical Oncology of Sun Yat-sen University Cancer Center, State Key Laboratory of Oncology in South China, Collaborative Innovation Center for Cancer Medicine, 651 Dongfengdong Road, Guangzhou, 510060 China; 2 Department of Medical Oncology and Guangdong Key Laboratory of Liver Disease, the Third Affiliated Hospital of Sun Yat-sen University, Guangzhou, China; 3 Department of pathology of Sun Yat-sen University Cancer Center, State Key Laboratory of Oncology in South China, Collaborative Innovation Center for Cancer Medicine, Guangzhou, China

**Keywords:** Colorectal cancer, Eukaryotic initiation factor 4A2 (EIF4A2), PDX, Silvestrol, ZNF143

## Abstract

**Background:**

Deregulation of protein translation control is a hallmark of cancers. Eukaryotic initiation factor 4A2 (EIF4A2) is required for mRNA binding to ribosome and plays an important role in translation initiation. However, little is known about its functions in colorectal cancer (CRC).

**Methods:**

Analysis of CRC transcriptome data from TCGA identified that EIF4A2 was associated with poor prognosis. Immunohistochemistry study of EIF4A2 was carried out in 297 paired colorectal tumor and adjacent normal tissue samples. In vitro and in vivo cell-biological assays were performed to study the biological functions of EIF4A2 on experimental metastasis and sensitivity to oxaliplatin treatment. Bioinformatic prediction, chromatin immunoprecipitation (ChIP) and dual-luciferase reporter assay were carried out to unveil the transcription factor of EIF4A2 regulation.

**Results:**

EIF4A2 Expression is significantly higher in colorectal tumors. Multivariate analysis suggests EIF4A2 as an independent predictor of overall, disease-free and progression-free survival. Dysfunction of EIF4A2 by genetic knock-down or small-molecule inhibitor silvestrol dramatically inhibited CRC invasion and migration, sphere formation and enhanced sensitivity to oxaliplatin treatment in vitro and in vivo. Notably, EIF4A2 knock-down also suppressed lung metastasis in vivo. qRT-PCR and immunoblotting analyses identified c-Myc as a downstream target and effector of EIF4A2. ChIP and dual-luciferase reporter assays validated the bioinformatical prediction of ZNF143 as a specific transcription factor of *EIF4A2*.

**Conclusions:**

EIF4A2 promotes experimental metastasis and oxaliplatin resistance in CRC. Silvestrol inhibits tumor growth and has synergistic effects with oxaliplatin to induce apoptosis in cell-derived xenograft (CDX) and patient-derived xenograft (PDX) models.

**Electronic supplementary material:**

The online version of this article (10.1186/s13046-019-1178-z) contains supplementary material, which is available to authorized users.

## Introduction

Colorectal cancer (CRC) is the second prevalent and third devastating cancer worldwide [1, 2]. In China, CRC is the third most common and fifth leading cause of cancer-related death [3]. Only 70.3% of stage II and 58.3% of stage III CRC patients survive longer than 5 years after curative therapies such as surgery, radiotherapy and chemotherapy [4]. In the era of immunotherapy, anti-programmed death 1 (PD-1) immune checkpoint inhibitors are currently only approved for metastatic CRC patients with dMMR (deficient DNA mismatch repair) status and many patients will eventually develop progressive disease (PD) due to drug resistance [5]. Drug resistance and metastasis remain to be the main causes of death in CRC patients. Therefore, it is necessary to identify genes affecting CRC drug resistance and metastasis.

Abnormality of mRNA translation often exists in malignant tumors, which contributes to tumor growth and metastasis [6]. Translation initiation is the most well-studied in cancer [7]. Translation initiation is regulated by eukaryotic translation factor 4F (EIF4F) heterotrimeric complex, which is consisted of EIF4A, EIF4E and EIF4G [8]. Eukaryotic translation initiation factor 4A (EIF4A) belongs to dead-box RNA helicase family and is the most abundant component among all the translation initiation factors. [9, 10]. The helicase activity of EIF4A affect different mRNA translation initiation rates and results in translation control of different mRNA [11]. In human, there are three family members of EIF4A: EIF4A1, EIF4A2 and EIF4A3 [12]. It has been reported that miR-133a plays a pivotal role in colorectal cancer by inhibiting cell proliferation, invasion, and migration by targeting oncogenic eukaryotic translation initiation factor 4A1 (EIF4A1) in colorectal cancer [13]. Interestingly, the translational target genes of EIF4A3, a close family member of EIF4A1, might be Cyclin D1, Cyclin E1 and CDK4, while the recruitment of EIF4A3 to these known oncogenes might be mediated by a novel long noncoding RNA H19, thus to affect the cell-cycle-regulatory gene expressions at the translational or post-translational level [14]. High EIF4A2 was also reported to be a prognostic factor of breast cancer and non-small cell lung cancer [15, 16]. However, little is known about function and regulatory mechanism of EIF4A2.

Here, we performed a series of cell-biological assays to explore the effects of knocking-down EIF4A2 on experimental metastasis and oxaliplatin resistance in colorectal cancer. We also explored on the specific transcription factor of *EIF4A2* and the effects of silvestrol [17] in CRC.

## Materials and methods

### Tissue samples and immunohistochemistry (IHC) analysis

Ethics committees of Sun Yat-sen cancer center approved our study. All patients included in our study provided written informed consent. We conducted this study and complied with the Helsinki declaration.

We obtained pathological slides and clinicopathological characteristics of 297 CRC patients hospitalized in Sun Yat-sen University Cancer Center from December 2006 to November 2012.

We conducted IHC procedures according to standard protocols as previously described [18]. Briefly, we incubated the tissue slides with anti-Ki67 (1:500) and anti-EIF4A2 (1:400) antibodies at 37 °C for 1 h. Subsequently, we incubated the tissue slides with biotinylated goat anti-rabbit immunoglobulin (1:75) at 37 °C for 30 min. Two experienced pathologists assessed the IHC staining in a double-blind way. They evaluated staining intensity of five representative fields and staining extent of each pathological section. The staining intensity scores ranged from 0 to 3 (0, negative staining; 1, weak staining; 2, moderate staining; 3, strong staining). The extent of EIF4A2-positive cells ranged from 0 to 100%, scoring from 0 to 3(0, < 25%; 1, 25–50%; 2, 50–75%; 3, 75–100%). We got the final score by adding these two scores for staining intensity and staining extent.

We used receiver operating characteristic curve (ROC) analysis defined the best cutoff value of EIF4A2 at 3.5. The EIF4A2 expression level was defined as high expression when the final score of EIF4A2>3.5. The EIF4A2 expression level was considered as low expression when the final score of EIF4A2 ≤ 3.5.

### Cell lines and cell culture

The human CRC cell lines DLD1, HCT116, HCT15, HCT8, RKO, Caco2, SW480, SW620, SW1463, SW837, HT29, CW2 and human colon epithelial cell line NCM460 were purchased from the American Type Culture Collection (Manassas, VA, USA). HCT116/Oxa (oxaliplatin-resistant HCT116 cell line) was bought from Oulu Biotechnology (Shanghai, China). All cell lines were authenticated by short tandem repeat DNA fingerprinting at the Medicine Lab of Forensic Medicine Department of Sun Yat-sen University (Guangzhou, China).

### Reagents and antibodies

The EIF4A2 inhibitor silvestrol was purchased from Med-Chemexpress (New Jersey, USA) and dissolved in DMSO. Oxaliplatin was purchased from Selleck Chemicals (Houston, TX, USA) and dissolved in water. The antibodies against the following proteins were used: EIF4A2, Ki67 and KRAS, MTA1, C-MYC (Abcam, Massachusetts, USA) and Vinculin (Cell Signaling Technology, Beverly, MA, USA).

### Lentivirus transfection

As previously described, the expression of EIF4A2 was knocked down by Short hairpin RNA (shRNA) directed against human EIF4A2, or a nontarget oligonucleotide was ligated into the LV-3 (pGLVH1/GFP + Puro) vector [19]. The lentivirus was synthesized by Obio Technology Corp. Ltd. (Shanghai, China). The HCT116 and DLD1 cells were infected with the lentivirus or control virus (NC) according to the manufacturer’s instructions. To obtain stably transfected cell lines, these cells were selected with puromycin (2 μg/mL) for 2 weeks. Knockdown efficiency was confirmed by qRT-PCR and western blot analysis. The stably transfected cells were classified as sh-CTRL (control), sh-*EIF4A2*–1 and sh-*EIF4A2*–2, which were used for subsequent experiments.

The sequences (5′ to 3′) were as follows:

- sh-CTRL: TTCTCCGAACGTGTCACGT;
- sh-*EIF4A2*–1: GCCAGAGACTTCACAGTTTCT;
- sh-*EIF4A2*–2: GCAACAAGTGTCTTTGGTTAT.

### RNA interference

The small interfering RNA (siRNA) duplex oligonucleotides targeting human ZNF143 mRNA, ZBTB33 mRNA, ATF4 mRNA, ETV4 mRNA and E2F6 mRNA were purchased from GenePharma (Guangzhou, China). Cells were transfected using Lipofectamine RNAiMAX (ThermoFisher Scientific, USA).

The siRNA sequences targeting *ZNF143* were as follows:

- si*ZNF143*–1: CAGAUGGUGACAACUUAGATT;
- si*ZNF143*–2: GGCAUUUCGAUGUGAAUAUTT;
- si*ZNF143*–3: GCCAGUGCAACAAAUUAUATT.

The siRNA sequences for *ZBTB33*, *ATF4*, *ETV4*, and *E2F6* were listed in the Additional file 1: Table S1.

### Apoptosis assays

Cell apoptosis were determined with flow cytometry as previously described [20]. Cell apoptosis induced by stably knockdown EIF4A2 in combination with oxaliplatin, silvestrol or oxaliplatin was determined by AnnexinV/PI or Annexin V/APC (KeyGEN, Nanjing, China). All these measurements were conducted with flow cytometry analysis (Beckman Coulter, USA).

### Quantitative real-time PCR (qPCR)

The levels of mRNA expression were measured by qRT-PCR in a LightCycler 480 instrument (Roche Diagnostics, Switzerland) as previously reported [21]. Briefly, sample RNA was extracted from cells by Trizol reagent (Life Technologies, USA) and then reversely transcribed to cDNA with a Takara kit (NHK, Japan). All samples were analyzed in a 10 μL volume system in triplicate. The gene expression was normalized using β-Actin as an internal reference, and the data were analyzed with the 2^-∆CT^ method. The specificity was verified by melting curve analysis.

Primers were synthesized by GENEray Biotechnology (Guangzhou, China). The primer sequences for real-time PCR were as follows:

- ① EIF4A2: TGGTGTCATCGAGAGCAACTG, GGCTTCTCAAAACCGTAAGCA;
- ② Beta-actin: TGGATCAGCAAGCAGGAGTA, TCGGCCACATTGTGAACTTT;
- ③ C-MYC: CGTCCTCGGATTCTCTGCTC, GATTTCTTCCTCATCTTCTTGTTC;
- ④ KRAS: ACAGAGAGTGGAGGATGCTTT, TTTCACACAGCCAGGAGTCTT;
- ⑤ MTA1: TTGTCTGTGAGTGGGTTGTGC, TGTTAAAAGAAGGCGAGGAGG.

Other sequences are listed in Additional file 1: Table S2.

### Western blotting analyses

We extracted protein by RIPA lysis buffer and quantified the protein by a BCA protein assay as previously described [22]. We separated the protein sample on 8–15% SDS-PAGE gels and then transferred them to polyvinylidene fluoride membranes (Immobilon-P, Millipore, Bedford, USA). We blocked the membranes with 5% non-fat milk in TBST for 1 h at room temperature and later incubated with the primary antibody at 4 °C overnight. In the second day we washed the membranes for three times with TBST and probed them with peroxidase-linked secondary antibody for 1 h at room temperature. Finally, we used enhanced chemiluminescence (SuperSignal ECL, ThermoFisher Scientific, USA) to visualize protein.

### Cell proliferation and colony formation assays

As previously described, Cell viability was tested with MTS assays (Qiagen, Germany) according to the manufacturer’s instructions and the colony formation assay as well [23]. In performing MTS assays, the absorbance was measured at a wavelength of 490 nm on a Synergy™ Multi-Mode Microplate Reader (Biotek, Vermont, USA). As for colony formation assays, 500 cells were seeded per well in 6-well plates. In assays testing therapeutic effect of silvestrol, silvestrol or control DMSO were added. After 14 days, the cells were fixed in methanol and stained with 0.2% crystal violet. The number of colonies was counted using Quantity One software (Bio-Rad, Hercules, CA, USA).

### Transwell migration and invasion assays

The effects of knocking-down EIF4A2 by shRNA or silvestrol on migration and invasion of CRC cells were tested by using transwell chambers as previously reported [24]. Briefly, cells were stably knocked-down EIF4A2 or pretreated with silvestrol for 24 h before the transwell assay, 200 μl of medium without FBS containing 1 × 10^5^ cells was added to the upper chamber, and 600 μl of 100% FBS was added to the lower chamber. Cells in the chambers were fixed with methanol and stained with crystal violet (Sigma-Aldrich, St. Louis, USA) after 24–48 h. Afterwards, the chambers were observed under a microscope and the migrated and invaded cells were counted.

### Sphere-forming assays

A sphere-forming assay was performed as previously described [25]. Briefly, 1.0× 10^3^ cells were seeded in 96-well plates (Corning Inc. Corning, USA) using the following medium: serum-free DMEM/F12 (Invitrogen) with 20 ng/mL of basic fibroblast growth factor (bFGF; Peprotech, Rocky Hill, NJ, USA), 1 × Penicillin-Streptomycin Solution, 20 ng/mL of epidermal growth factor (EGF; Peprotech),10 μg/mL of heparin (Sigma-Aldrich, St. Louis, USA) and 1% B-27 (Life Science). 1 week later, the size and number of tumor spheres were evaluated.

### Chromatin immunoprecipitation (ChIP) assays

The procedure of ChIP was performed as described previously [26]. The cells (4 × 10^6^) were cross-linked by using 1% paraformaldehyde and used for each immunoprecipitation experiment. ZNF143 antibody (Abcam, Massachusetts, USA) or the isotype-control antibody (rabbit IgG, Abcam) was used. PCR and real-time quantitative PCR using specific primers of EIF4A2 promotor were performed to identify the precipitated DNA. The signals were calculated as the percentage of input.

### Dual-luciferase reporter assay

Dual-Luciferase Reporter Assay was performed as previously described [27]. PGL4.10EIF4A2 promotor (wild type), PGL4.10EIF4A2 promoter (mutant), pcDNA3.1-ZNF143–3FLAG, PGL4.10 (Control plasmid) and renilla plasmid were synthesized by Oobio Technology Corp., Ltd. (Shanghai, China). Bioinformatics analysis showed that the sequence of binding site of EIF4A2 promotor was TTTTGCACCATGGGTA, the region was -1208 bp to -1192 bp. Briefly, the cells were seeded to the 96-well plate at 70% confluence. Each sample with 6 duplicated holes. After 16 h, the plasmids were transfected using Lipofectamine 2000(lipo2000). The ratio of plasmids and lipo2000 per reaction was: plenti: firefly: renilla: lipofectamine 2000 = 0.2 μg: 0.1 μg: 0.01 μg: 0.25 μl. After 48 h, a luciferase reporter assay was carried out by the Dual-Luciferase Reporter Assay System (Promega, Madison, WI, USA). The renilla and firefly luciferase activity were evaluated.

### TdT-mediated dUTP nicked-end labeling (TUNEL) assay

The cell death detection kit (Biotool, Houston, TX, USA) was used to perform TUNEL assay [28]. Briefly, we dewaxed, rehydrated tissue sections and incubated them with proteinase K and specific probes. We used DAPI (Invitrogen, Carlsbad, USA) to counterstain the nuclei and next mounted the tissue section with ProLong Gold antifade reagent (Invitrogen, Carlsbad, USA). We randomly selected three fields to count the number of TUNEL positive cells and we took representative images with an Olympus FV1000 microscope (Olympus, Tokyo, Japan).

### Establishment of patient-derived xenograft (PDX) model

The procedures were performed as previously described [29]. Briefly, we put the fresh tumor samples from CRC patients receiving surgery in our cancer center in a sterile tube containing cold culture medium. We cut the tumor samples into several equal pieces and subcutaneously implanted into dorsal flank of Biocytogen-NOD-SCID-IL2rg (BNDG) mice (Biocytogen Co., Ltd., Jiangsu, China) within 8 h after the surgery. We measured the size of tumor every 3 days. We nominated the mice with successfully established PDXs as passage 1 (P1). We removed the tumors and cut them into several equal pieces and implanted subcutaneously into another BNSG mouse to get the next generation (P2) when the tumor volume reached about 600 mm3. We used the mice with P2 PDX to test the efficacy of silvestrol, oxaliplatin and the combination. PBS, Silvestrol or Oxaliplatin was injected every 3 days. Tumor diameter, tumor width and body weight of the mice were measured every 3 days. The mice were sacrificed 4 weeks later. IHC was used to test the expression of Ki67. The TUNEL assays were used to detect number of TUNEL-positive cells.

### Establishment of cell-derived xenograft (CDX) model and in vivo therapeutic study

The procedures were performed as previously described [29]. HCT116 and DLD1 cells, with or without stably knockdown of EIF4A2 were used to establish the CDX. The cells (2 × 10^6^) were suspended in 50 μL cold PBS + 50 μL Matrigel (BD biocoat) and injected subcutaneously into the dorsal flank of the 4-week-old BALB/c nude mice (Beijing Vital River Laboratory Animal Technology Co., Ltd). There were two groups: Sh-CTRL treated with oxaliplatin and Sh-EIF4A2–1 treated with oxaliplatin (5 mg/kg every 3 days). The mice were sacrificed 4 weeks later. IHC was used to test the expression of Ki67. The model of pulmonary metastasis by tail vein injection as follows: 1 × 10^6^ cells were suspended in 100 μL cold PBS and injected into the tail vein of mice. The mice were sacrificed after 2 months. Metastatic nodules in the lung were measured.

The HCT116 and DLD1 CDX, PDX were used to test the efficacy of silvestrol, oxaliplatin and the combination. We randomly assigned the mice to four groups: (1) The control group, which received 200 ml of PBS;(2) The oxaliplatin group, which received Oxaliplatin at 5 mg/kg by i.p. injection; (3) The silvestrol group, which received silvestrol at 1.5 mg/kg by i.p. injection; (4) The combination group. PBS, Silvestrol or Oxaliplatin was injected every 3 days. Tumor diameter, tumor width and body weight of the mice were measured every 3 days. The mice were sacrificed 4 weeks later. IHC was used to test the expression of Ki67. The TUNEL assays were used to detect number of TUNEL-positive cells.

### Statistical analysis

Each cellular experiment was repeated at three biological replicates. The cutoff value of EIF4A2 was defined using receiver operating characteristic (ROC) curve analysis. The correlations between ZNF143 mRNA and EIF4A2 mRNA were analyzed by Pearson correlation test. We compared differences of continuous variable by Student’s t-tests. We compared differences of categorical factors between groups by Chi-square test and Fisher’s exact test. We compared median values between different groups by Mann-Whitney test. Propensity scores for 297 patients were estimated by a logistic regression model using the following factors as covariates: age, gender, lymph node metastasis, nerve invasion, invasion depth, vascular thrombus, distant metastasis status, degree of differentiation and TNM stage. Finally, 81 pairs of patients were generated by a one-to-one nearest-neighbor matching algorithm with an optimal caliper of 0.2 without replacement. In univariate analyses, we estimated survival difference of distinct variables using Kaplan–Meier method (log-rank test) and we subsequently identified independent prognostic factors by Cox proportional hazards regression models in multivariate analysis. All the tests were two-tailed and *P* < 0.05 was considered statistically significant. Statistical analysis was performed by using Graphpad Prism 6.0(GraphPad Software Inc., La Jolla, CA, USA) and R statistical package (R software version 3.4.1; R Foundation for Statistical Computing, Vienna, Austria).

## Results

### EIF4A2 is upregulated in CRC and predicts poor survival of patients

**Fig. 1:**
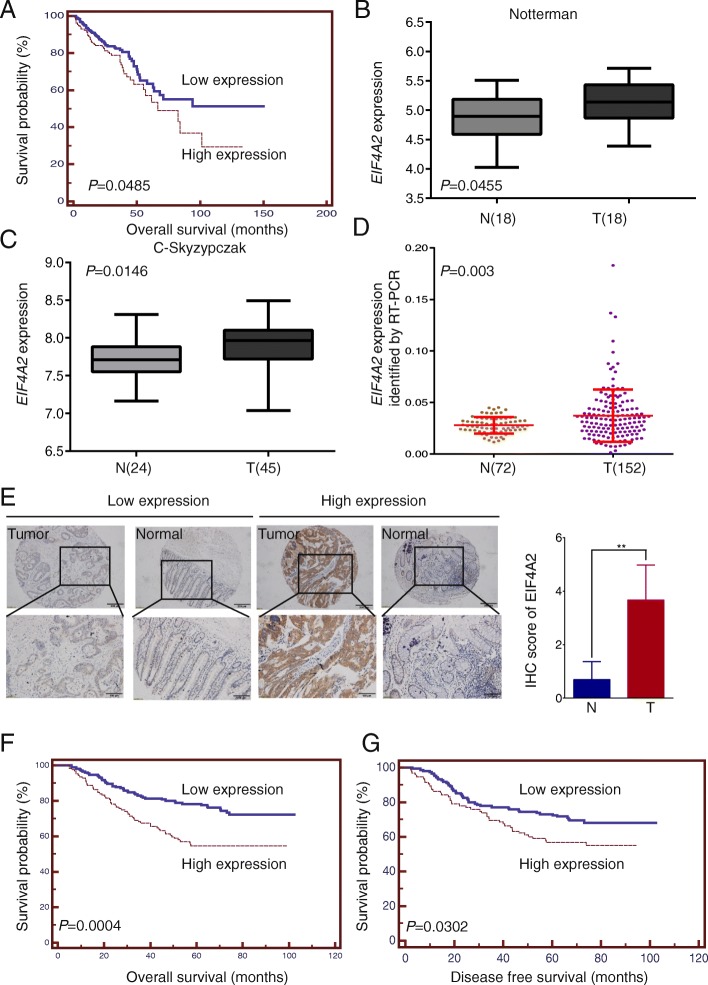
Expression of EIF4A2 is high in colorectal tumors and predicts poor survival of patients. **a** Data analysis of TCGA identified high *EIF4A2* level was associated with poor prognosis in CRC patients. **b**, **c** The EIF4A2 mRNA level was significantly higher in CRC tissues than that of normal tissues in Notterman dataset and C-skyzypczak dataset from Oncomine (https://www.oncomine.com). **d** The *EIF4A2* level was significantly higher in 152 human CRC tissues that of 72 normal tissues hospitalized in SYSUCC. **e** Representative images show low or high expression of EIF4A2 in CRC tumor tissues compared to adjacent normal tissues analyzed by IHC. The EIF4A2 expression was significantly higher in tumor tissues (**, *P* < 0.01). **f** The overall survival curve of 297 CRC patients with low and high expression of EIF4A2 were generated using the Kaplan-Meier method (log-rank test). **g** The disease-free survival curve of 245 CRC patients underwent curative surgery with low and high expression of EIF4A2 were generated using the Kaplan-Meier method (log-rank test)

We analyzed TCGA patient data and observed that high EIF4A2 expression was associated with poor prognosis (Fig.1a). In contrast to EIF4A2, high EIF4A3 was associated with better prognosis, whereas EIF4A1 showed no correlation (Additional file 1: Figure S1A-B). We further investigated EIF4A2 expression profile in human CRC. Consistently, expression of EIF4A2 was significantly higher in CRC tissues in Notterman and C-skyzypczak data sets and our own CRC patient cohort on both mRNA and protein level (Fig.1b-d, Additional file 1: Figure S1C-D).

The EIF4A2 protein expression in 297 human CRC specimens was analyzed by IHC and patients’ clinicopathological variables were also collected to further identify the clinical significance of EIF4A2 (Table 1). IHC results showed that EIF4A2 expression was significantly higher than that in adjacent normal tissues (Fig.1e). Notably, high expression of EIF4A2 was significantly correlated with more distant metastasis and TNM stage IV (Table 1) and associated with much shorter overall survival (OS) of 297 patients, disease-free survival (DFS) of 245 patients with curative surgery and progression free survival (PFS) of 52 metastatic CRC patients (Fig.1f, Additional file 1: Figure S1E). As we can see in the Table 1, 23.2% of patients in the high EIF4A2 expression group were with distant metastasis, while only 13.4% of patients in the low EIF4A2 expression group were with distant metastasis. Multivariate analysis further suggested that EIF4A2 could be used as an independent predictor of OS, DFS, and PFS (Table 2, Additional file 1: Table S3 and S4).

**Table 1:** Baseline characteristics and Correlation analysis for clinicopathologic variables between EIF4A2-low and EIF4A2-high groups of 297 CRC patients

Variable	Total (%)	low EIF4A2 n (%)	high EIF4A2 n (%)	*P* value
Total	297 (100)	172 (57.9)	125 (42.1)	
Age, years				0.557
> 57	137(46.1)	82 (47.7)	55 (44.0)	
≤ 57	160(53.9)	90 (52.3)	70 (56.0)	
Gender				0.469
male	185 (62.3)	104 (60.5)	81 (64.8)	
female	112(37.7)	68 (39.5)	44 (35.2)	
Lymph node metastasis				0.184
N0–1	218(73.4)	121 (69.2)	97 (77.6)	
N2	79(26.6)	51 (30.8)	28 (22.4)	
Nerve invasion				0.078
No	152 (51.2)	96 (55.8)	56 (44.8)	
Yes	145 (48.8)	76 (44.2)	69 (55.2)	
Invasion depth				0.115
No whole layer	29 (9.8)	21 (12.2)	8 (6.4)	
Whole layer	268 (90.2)	151 (87.8)	117 (93.6)	
Vascular thrombosis				0.252
No	206 (69.4)	124 (72.1)	82 (65.6)	
Yes	91 (30.6)	48 (27.9)	43 (34.4)	
Metastasis status				0.031
No	245 (82.5)	149 (86.6)	96 (76.8)	
Yes	52 (17.5)	23 (13.4)	29 (23.2)	
Degree of differentiation				0.674
Well and moderately differentiated	230 (77.4)	135 (78.5)	95 (76.0)	
Poorly differentiated	67 (22.6)	37 (21.5)	30 (24.0)	
TNM stage				0.003
I	20 (6.7)	12 (7.0)	8 (6.4)	
II	87 (29.3)	42 (24.4)	45 (36.0)	
III	138 (46.5)	95 (55.2)	43 (34.4)	
IV	52 (17.5)	23 (13.4)	29 (23.2)

**Table 2:** Univariate and multivariate analyses of prognostic factors for OS of 297 patients with colorectal cancer

Variable	Univariate		Multivariate				
	Log-rank χ^2^	*P* value	B	SE	HR	95% CI	*P* value
Gender (male/female)	3.425	0.064					
Age, years (> 57/≤ 57)	1.320	0.251					
Lymph node metastasis (Yes/No)	18.235	< 0.001	0.645	0.400	1.907	0.871 to 4.173	0.106
Vascular thrombosis (Yes/No)	69.372	< 0.001	0.689	0.300	1.993	1.106 to 3.589	0.022
Pathology differentiation (Well and moderately differentiated/poor differentiation)	6.978	0.008	0.685	0.239	1.931	1.209 to 3.083	0.006
TNM (AJCC 7th) (I-II/III-IV)	25.524	< 0.001	0.078	0.513	0.925	0.339 to 2.527	0.879
Invasion depth (Whole layer/ no whole layer)	5.369	0.021	0.270	0.527	1.310	0.466 to 3.60	0.609
Nerve invasion (yes /no)	35.229	< 0.001	0.505	0.253	1.657	1.010 to 2.718	0.045
Metastasis status (yes/no)	75.425	< 0.001	0.953	0.314	2.594	1.403 to 4.796	0.002
EIF4A2(high expression/low expression)	12.750	< 0.001	0.597	0.211	1.816	1.201 to 2.747	0.005

*OS* overall survival

Interestingly, TCGA patient data analysis also showed that EIF4A2 expression was prognostic of poor prognosis in liver cancer, head and neck cancer, melanoma and prostate cancer (Additional file 1: Figure S1F-G).

To reduce the confounding bias in baseline characteristics [30], we carried out propensity score matching (PSM) between high EIF4A2 group and low EIF4A2 group (Additional file 1: Table S5). Univariate analysis showed that EIF4A2 expression, lymph node metastasis, vascular thrombosis, pathology differentiation, TNM stage, nerve invasion, distant metastasis status were prognostic factors of OS. Multivariate analysis showed that nerve invasion and EIF4A2 expression were independent prognostic factors of OS (Additional file 1: Table S6).

To further create a more accurate prediction model, we established a prognostic nomogram including EIF4A2, distant metastasis, pathology grade, nerve invasion and vascular thrombosis in 297 CRC patients (Additional file 1: Figure S2A). Kaplan-Meier analyses showed that the nomogram was able to significantly distinguish the prognosis of CRC patients in different groups (*P* < 0.0001) (Additional file 1: Figure S2B). The AUC (area under curves) of the Nomogram was significantly larger than that of the TNM staging system (0.773 vs 0.710, *P* = 0.0269) (Additional file 1: Figure S2C).

### Knocking-down EIF4A2 inhibits migration and sphere formation of CRC cells

**Fig. 2:**
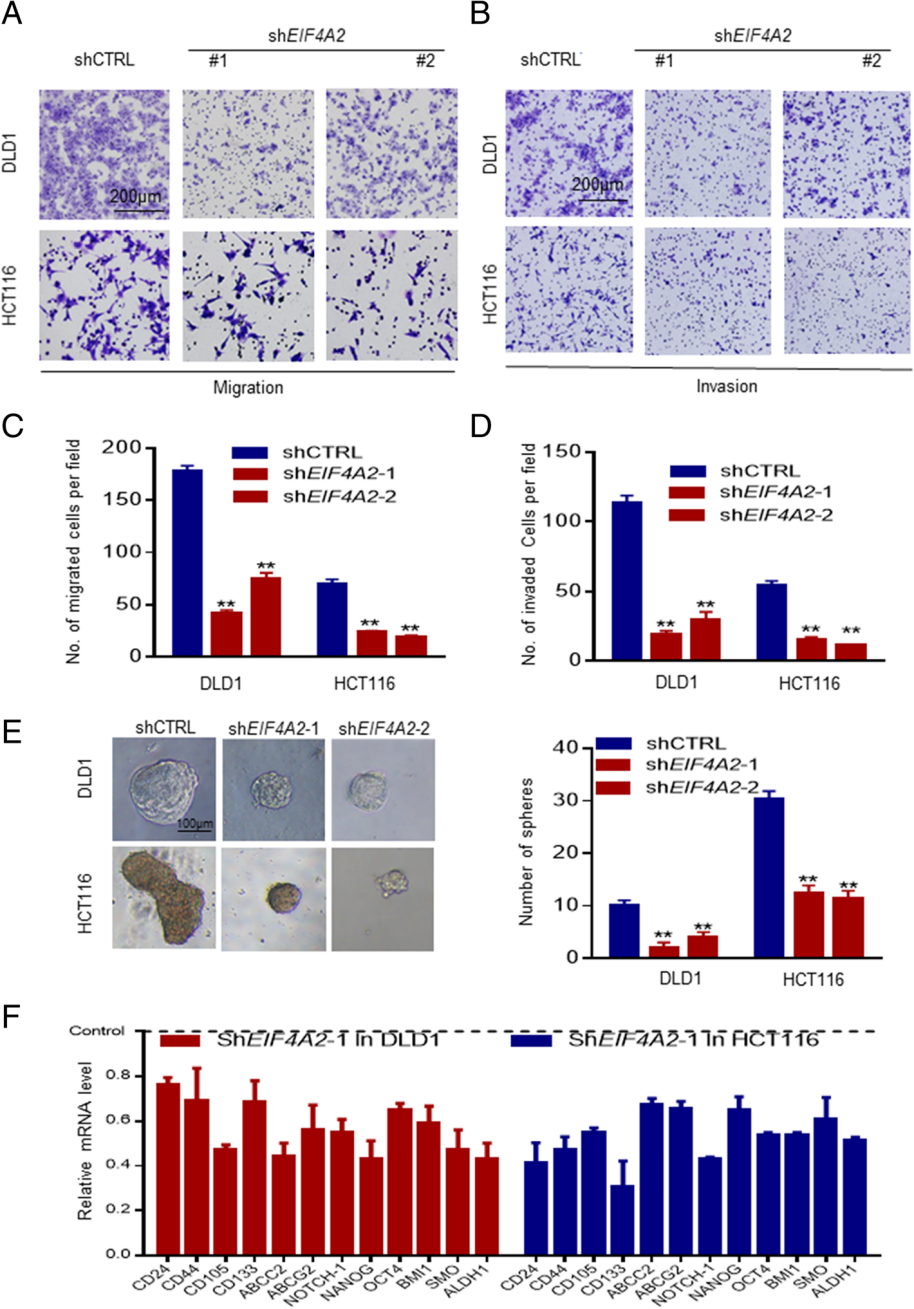
Knocking-down *EIF4A2* inhibits migration and invasion of CRC cells. **a**, **b**, **c**, **d** Knocking-down *EIF4A2* significantly inhibited transwell migration and invasion in DLD1 and HCT116 cells. **e** Knocking-down *EIF4A2* significantly reduced sphere formation in DLD1 and HCT116 cells. **f** Knocking-down *EIF4A2* significantly reduced a panel of stemness-associated genes (*NANOG, OCT-4, BMI-1, NOTCH-1, ALDH1 and SMO*), surface antigens associated with cancer stem cells (*CD24*, *CD44*, *CD105* and *CD133*) and cell surface transporter genes (*ABCC2* and *ABCG2*) related to drug resistance in DLD1 and HCT116 cells. *, *P* < 0.05; **, *P* < 0.01 versus the control

Given the prognostic value of EIF4A2 in colorectal cancer, we examined next whether EIF4A2 could serve as a therapeutic target. Compared to the normal human epithelial cell line NCM460, expression of EIF4A2 was significantly higher in CRC cell lines both at mRNA and protein level (Additional file 1: Figure S3A-B). Lentiviral infection of shRNA targeting EIF4A2 in DLD1 and HCT116 cells significantly inhibited cell migration and invasion (Additional file 1: Figure S2A-D, Figure S3C-D). Knockdown of EIF4A2 also strongly reduced sphere formation (Fig. 2e). Furthermore, qRT-PCR showed significantly reduced mRNA transcription of a panel of stemness-associated genes (*NANOG, OCT-4, BMI-1, NOTCH-1, ALDH1, and SMO*), cancer stem cells-associated surface antigens (*CD24*, *CD44*, *CD105,* and *CD133*) and multiple drug-resistant transporter genes (*ABCC2* and *ABCG2*) in EIF4A2 knocked-down cells (Fig. 2f).

### Knocking-down EIF4A2 inhibits lung metastasis in vivo

**Fig. 3:**
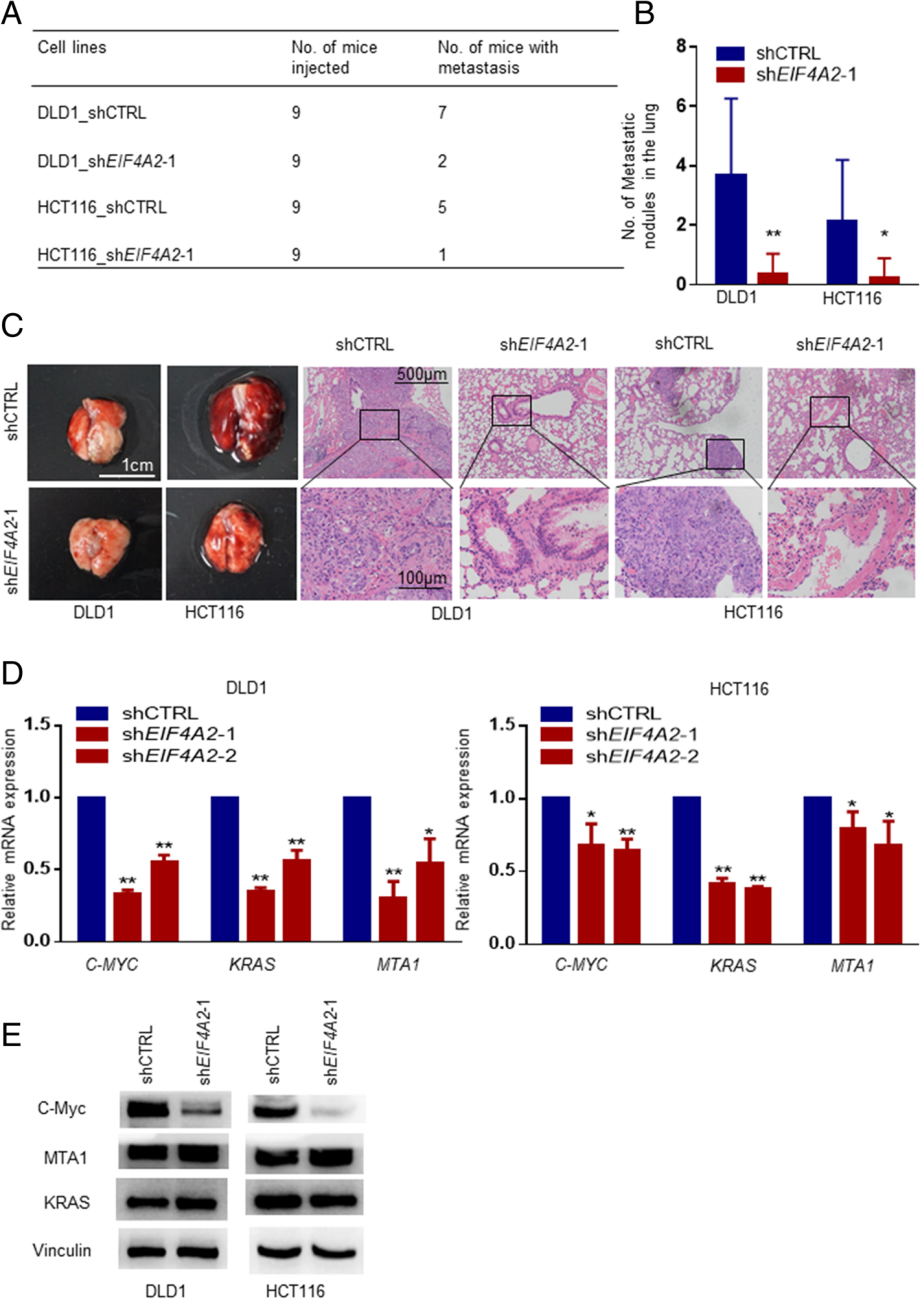
Knocking-down *EIF4A2* inhibits metastasis formation in the lung. **a**, **b** Knocking-down *EIF4A2* significantly reduced numbers of mice with lung metastasis (**a**) and metastatic nodules (**b**). **c** Representative images of HE staining showed metastatic nodules. **d** RT-qPCR arrays were used to screen 84 metastasis-related genes and showed that *C-MYC*, *KRAS* and *MTA1* mRNAs significantly reduced after knocking-down *EIF4A2*. **e** Western blots showed that c-Myc significantly decreased after knocking-down *EIF4A2* stably, but MTA1 and KRAS remained unchanged. *, *P* < 0.05; **, *P* < 0.01 versus the control

To investigate the effect of EIF4A2 on lung metastasis in vivo, HCT116 and DLD1 cells with or without stable knockdown of EIF4A2 were injected into mice. As a result, cells with shEIF4A2 had a much lower proportion of lung metastasis. 77.8% (DLD1) and 55.6% (HCT116) of mice with shCTRL cells formed lung metastasis, while only 22.2% (DLD1) and 11.1% (HCT116) of mice with shEIF4A2–1 cells formed lung metastasis (Fig. 3a). Moreover, the numbers of metastatic nodules in the lungs were significantly reduced in mice injected with DLD_shEIF4A2–1/ HCT116_shEIF4A2–1 cells compared with the numbers in those injected with DLD1_shCTRL/ HCT116_shCTRL cells (Fig. 3b and c).

To further identify downstream factors of EIF4A2 in CRC metastasis, qRT-PCR array containing 84 metastasis-related gene probes (Additional file 1: Table S7) was used to compare mRNA expression profiles of cells with shEIF4A2–1 to cells with shCTRL [31]. Interestingly, C-MYC, KRAS and MTA1 were downregulated on transcriptional level with shEIF4A2–1 (Fig. 3d). Immunoblotting result showed consistently reduced C-MYC protein level in shEIF4A2–1 cells, while KRAS and MTA1 remained unchanged (Fig.3e).

### Knocking-down EIF4A2 sensitizes CRC cells to Oxaliplatin treatment

**Fig. 4:**
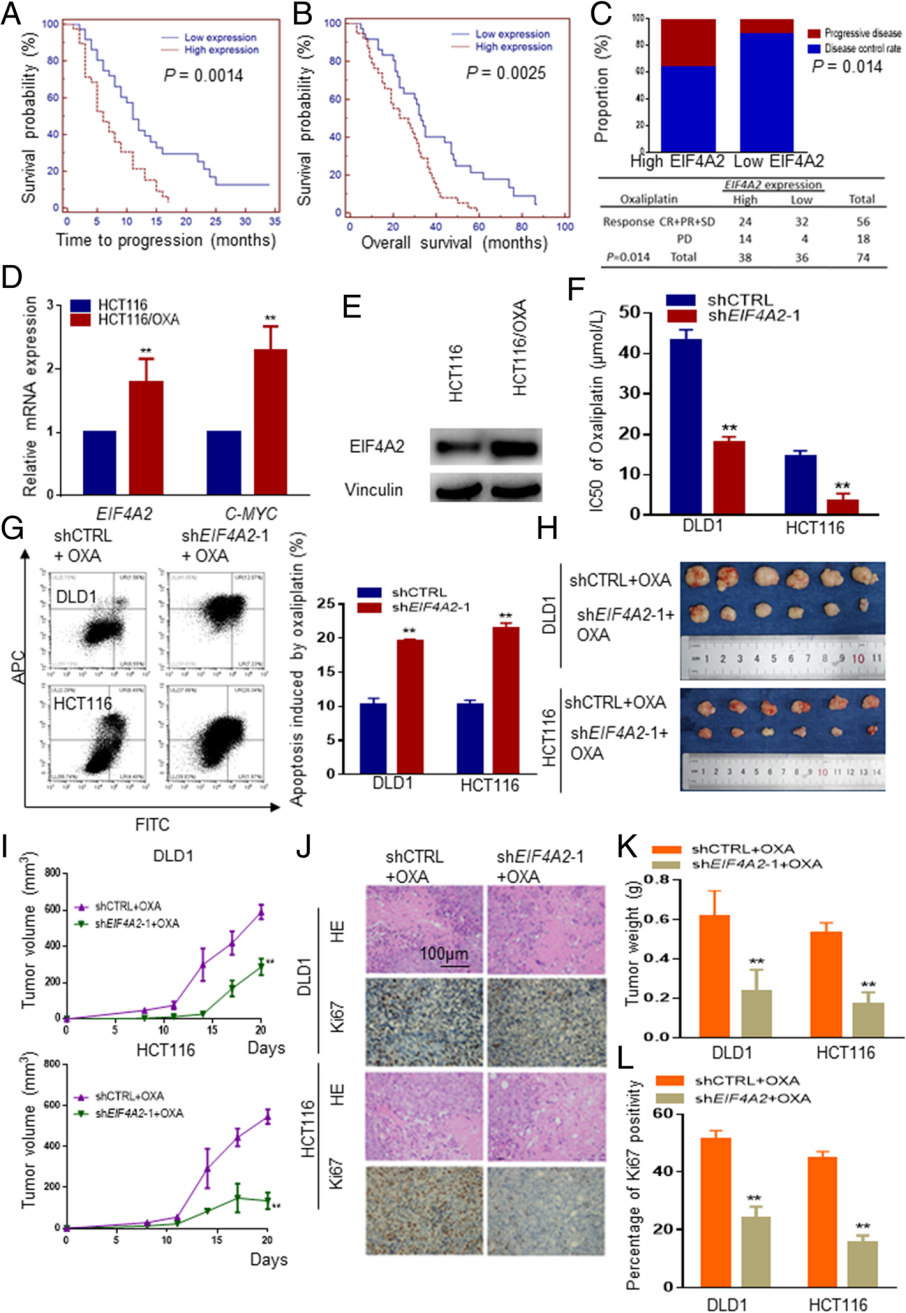
Knocking-down *EIF4A2* improves sensitivity of CRC cells to oxaliplatin. **a**, **b** IHC was performed to quantify the expression of EIF4A2 in 74 advanced CRC patients receiving oxaliplatin-based regimens as the first-line chemotherapy. Time to progression and overall survival were generated by the Kaplan-Meier method (log-rank test). **c** High EIF4A2 expression indicated poor response to oxaliplatin-based regimens. 36.8% of CRC patients with high EIF4A2 expression were evaluated as PD, while 11.1% of CRC patients with low EIF4A2 expression were PD. **d** *EIF4A2* and *C-MYC* mRNA levels significantly increased in HCT116/OXA cells. **e** Western blots showed that EIF4A2 obviously increased in HCT116/OXA cells. **f** The half-maximal inhibitory concentration (IC50) of oxaliplatin significantly decreased in HCT116 and DLD1 cells stably knocking-down *EIF4A2*. **g** The apoptosis rate induced by oxaliplatin in HCT116 and DLD1 cells with *EIF4A2* knockdown was significantly higher. **h**, **i**, **k** The sh*EIF4A2*–1 stably transduced cells treated with oxaliplatin showed the most significant reduction in tumor weight and volume. **j**, **l** IHC staining showed that the number of KI67-positive cells decreased most significantly in the sh*EIF4A2*–1 stably transduced cells treated with oxaliplatin. *, *P* < 0.05; **, *P* < 0.01 versus the control

Oxaliplatin resistance is one of the common causes of treatment failure in advanced CRC patients [32]. To determine whether EIF4A2 contributes to oxaliplatin resistance in CRC, IHC was used to analyze samples of 74 CRC patients receiving oxaliplatin-based regimens as first-line chemotherapy. Overall survival and time to progression were significantly shorter in CRC patients with high EIF4A2 expression than that of CRC patients with low EIF4A2 expression (Fig. 4a and b). Furthermore, 36.8% of patients with high EIF4A2 expression was evaluated as progressive disease (PD), while only 11.1% of patients with low EIF4A2 expression was evaluated as PD when receiving oxaliplatin based regimens as first-line chemotherapy (Fig.4c). To further clarify the expression of EIF4A2 in oxaliplatin-resistance, we analyzed the expression of EIF4A2 in oxaliplatin-resistant HCT116 cells (HCT116/OXA). qRT-PCR showed higher EIF4A2 and C-MYC mRNA level in HCT116/OXA cells than that in HCT116 (Fig.4d), and immunoblotting analysis showed consistent higher EIF4A2 protein level (Fig.4e). These results imply the potential role of EIF4A2 in oxaliplatin-resistance.

When CRC cells with EIF4A2 knockdown were treated with oxaliplatin, the IC50 value was significantly lower and inhibition rate was significantly increased compared to the control group (Fig.4f). Moreover, the apoptosis rate induced by oxaliplatin in CRC with EIF4A2 knockdown was significantly higher than that in the control group (Fig.4g).

To confirm the effects of EIF4A2, we overexpressed EIF4A2 in the DLD1 and HCT116 cell lines with stably knocking-down EIF4A2, which were named as sh-EIF4A2–1 + OE and sh-EIF4A2–2 + OE. We found that the migration ability, invasion ability and IC50 of oxaliplatin of sh-EIF4A2–1 + OE and sh-EIF4A2–2 + OE cell lines recovered to the level of the control group (Additional file 1: Figure S4A-G).

To determine whether knocking down EIF4A2 affects oxaliplatin sensitivity in vivo, CRC cells with shEIF4A2–1 or shCTRL were subcutaneously injected into nude mice and treated with oxaliplatin. Knocking-down EIF4A2 combined with oxaliplatin inhibited tumor volume and tumor weight significantly than that of shCTRL combined with oxaliplatin (Fig. 4h, i and k). ShEIF4A2 combined with oxaliplatin also have lower percentage of Ki67-positive cells (Fig. 4j and l).

### Silvestrol suppresses tumor progression and increases sensitivity to oxaliplatin in CRC cells

**Fig. 5:**
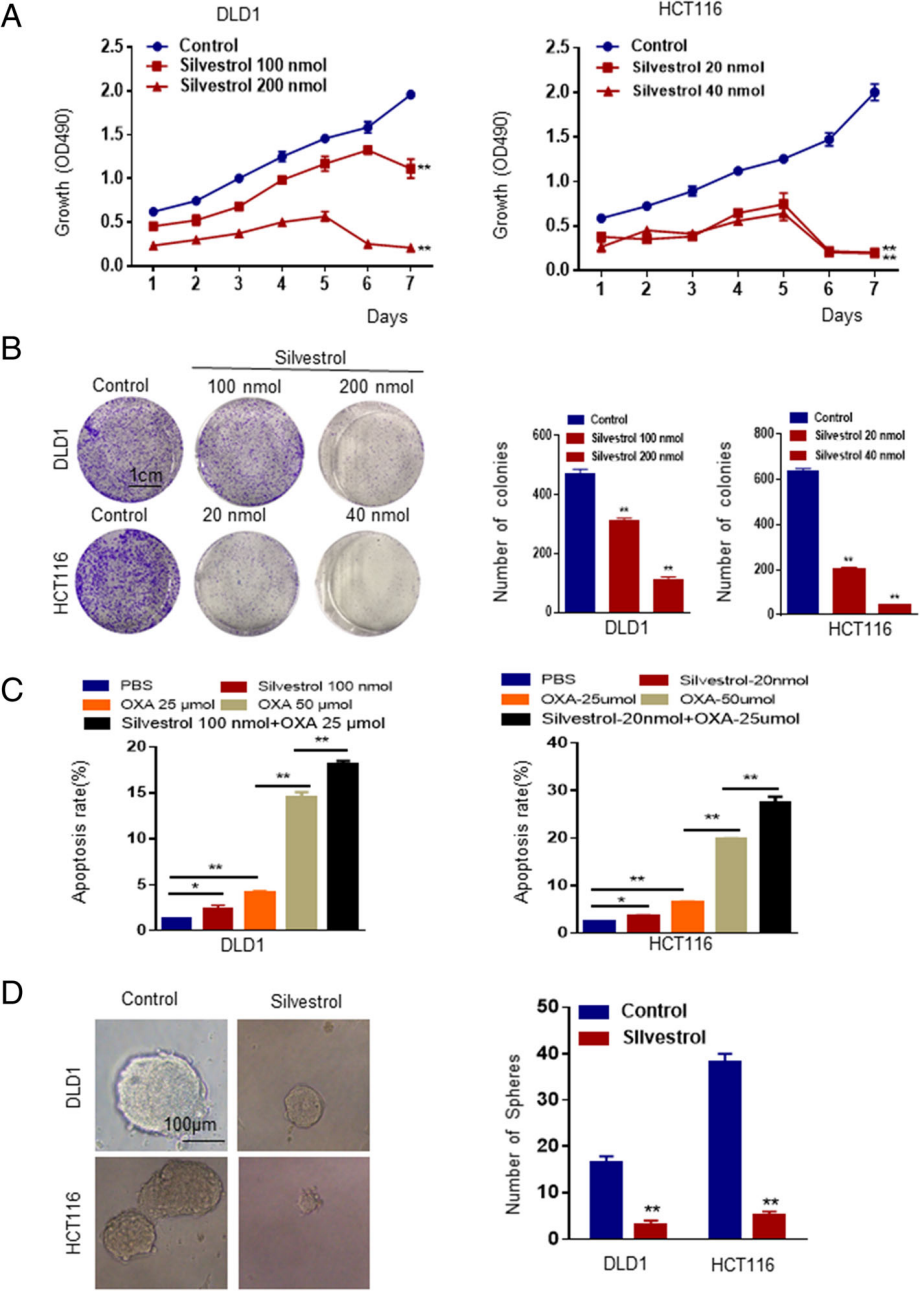
EIF4A inhibitor Silvestrol inhibits tumor growth, sphere formation and induces apoptosis when combined with oxaliplatin in CRC cells. **a** MTT assays showed Viability of the DLD1 and HCT116 cells treated with silvestrol of different concentrations. **b** Images (left) and quantification (right) of the indicated cells treated with silvestrol in colony formation assays. **c** Quantification of cell apoptosis of the indicated cells treated with silvestrol in Annexin-V/propidium iodide (PI) assays. **d** Representative images (left) and quantification (right) of sphere formation of the indicated cells treated with silvestrol. *, *P* < 0.05; **, *P* < 0.01 versus the control

Silvestrol is a small molecule extracted from *Aglaia foveolata*. Silvestrol can inhibit protein translation initiation by targeting EIF4A, including EIF4A1 and EIF4A2 [15]. Currently, there is no inhibitor specifically targeting EIF4A2. To test its effect in CRC, we treated DLD1 and HCT116 cells with silvestrol. MTS assays showed that cell growth was significantly inhibited in a time-dependent and concentration-dependent manner (Fig. 5a). Silvestrol also inhibited colony formation, migration, invasion and sphere formation of cancer cells (Additional file 1: Figure S5B, S5D, S5A). Intriguingly, combination treatment with oxaliplatin and silvestrol dramatically induced apoptotic rate in cancer cells compared to single drug treatment (Additional file 1: Figure S5C and S5B).

**Fig. 6:**
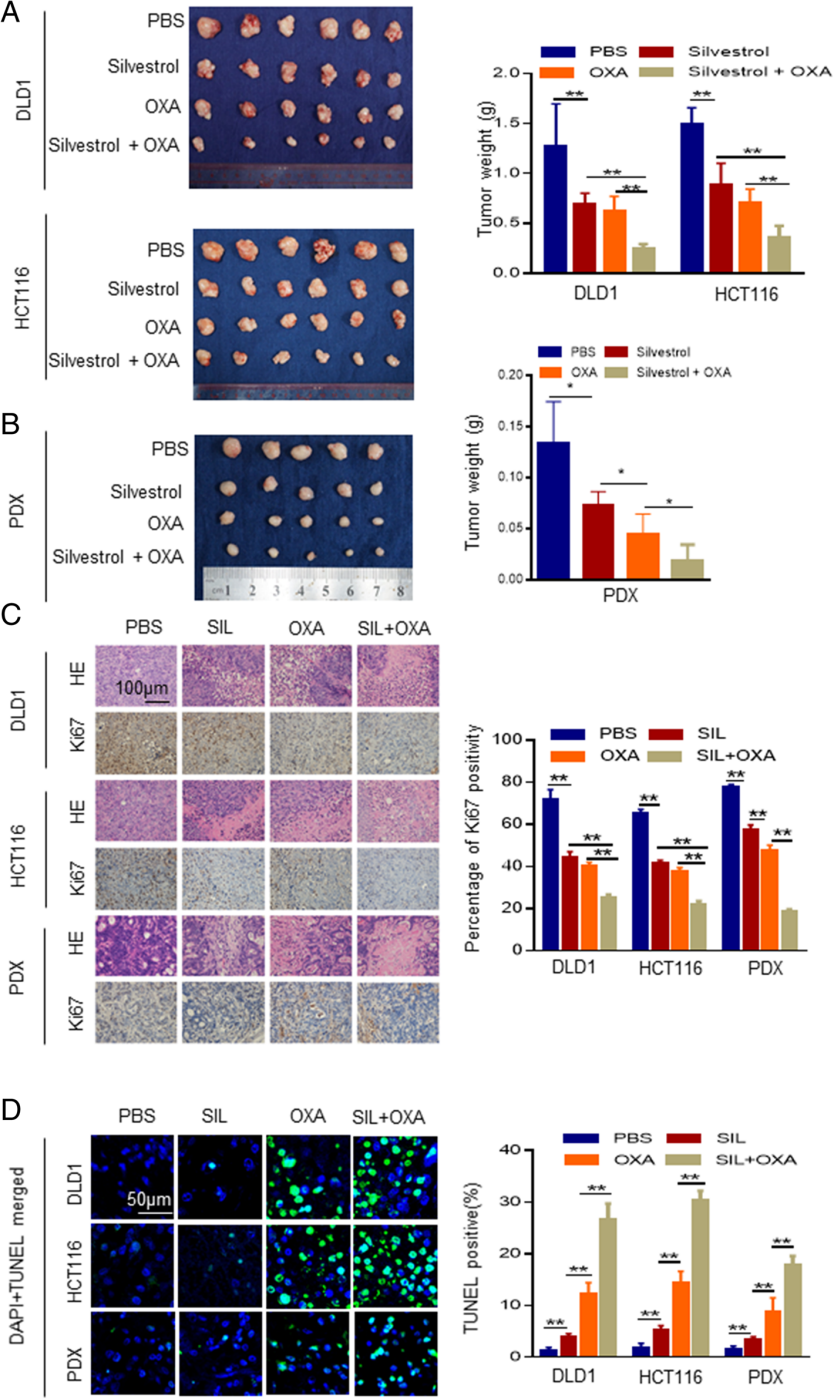
Silvestrol (SIL) inhibits tumor growth and has synergistic effects with oxaliplatin (OXA) in CDX and PDX. **a**, **b** Mice planted with indicated cancer cells or PDX were treated as indicated. The group treated with SIL and OXA together showed the most significant decrease in tumor volume and weight in the CDX (**a**) and PDX (**b**) models. **c** The mice treated with SIL + OXA combination treatment showed the most significant decrease of Ki67-positive cells in IHC staining. **d** The mice treated with SIL + OXA combination treatment showed the most significant increase of apoptotic cells in TUNEL assays. *, *P* < 0.05; **, *P* < 0.01 versus the control

We further verified the therapeutic efficacy of silvestrol with or without oxaliplatin in both CDX and PDX models. Consistent with our observation in vitro, xenograft tumors treated with silvestrol and oxaliplatin in combination experienced the most remarkable decrease in tumor volume and tumor weight in both CDX and PDX models (Fig. 6a and b). Ki67 IHC staining and TUNEL staining indicated the most prominent proliferation suppression and apoptosis induction in the combination treatment group (Fig. 6c and d). No significant body weight loss was observed in the experimental animals. Our results suggested that the EIF4A inhibitor silvestrol has therapeutic value in advanced CRC and synergistic effect with oxaliplatin, which might be worthy of clinical trials to further confirm the optimal dose and the efficacy in CRC patients.

### Transcription of EIF4A2 is regulated by ZNF143

**Fig. 7:**
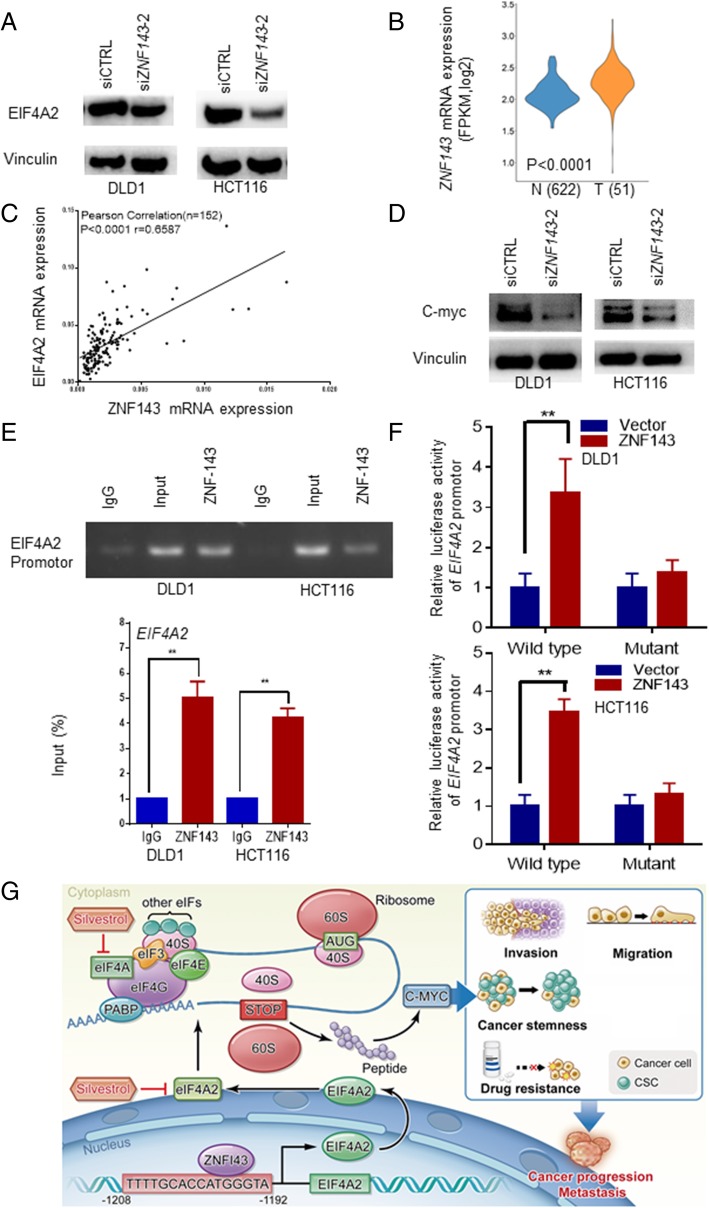
Transcription of *EIF4A2* is regulated by ZNF143. **a** Western blot analyses showed that EIF4A2 were significantly decreased after si*ZNF143*–2 transfection in the indicated cells. **b** Level of *ZNF143* was significantly higher in tumors than in normal tissues of TGCA CRC dataset. **c** A positive correlation was found between the mRNA levels of *ZNF143* and *EIF4A2*. **d** Western blot analyses showed that c-Myc were significantly decreased after si*ZNF143*–2 transfection in the indicated cells. **e** ChIP assays confirmed that ZNF143 could bind to the EIF4A2 promoter in DLD1 and HCT116 cells. Quantification of immunoprecipitated DNA was shown by RT-qPCR. **f** Luciferase assays showed that ZNF143 overexpression increased the luciferase activities driven by *EIF4A2* promoter, while co-transfected point-mutated *EIF4A2* promoter showed no response. **g** EIF4A2 promotes experimental metastasis and oxaliplatin resistance in CRC. ZNF143 is transcription factor of EIF4A2. Silvestrol inhibits tumor growth, invasion, migration, cancer stemness and improves oxaliplatin resistance. *, *P* < 0.05; **, *P* < 0.01 versus the control

The MSK-IMPACT analysis showed the proportion of EIF4A2 DNA amplification in CRC was only 0.8% [33], while our study found that EIF4A2 mRNA and protein expression in colorectal cancer was up to approximately 40%. Therefore, we speculated that EIF4A2 expression might be regulated at the transcription level. To further clarify the transcription regulators of EIF4A2, bioinformatics analysis was performed. After a series of analyses including binding motif analysis and co-expression analysis, the following 5 transcription factors were considered as possible transcription factors: ETV4, E2F6, ZBTB33, ATF4 and ZNF143. Next, we used siRNA approach to selectively knock-down their expression and observed that EIF4A2 expression decreased when knocking-down ZNF143 both in cancer cells (Fig. 7a). ZNF143 expression was significantly higher in CRC tumors in both TGCA data and our own samples (Additional file 1: Figure S7B and S6A), in which ZNF143 and EIF4A2 transcription is tightly correlated (Fig. 7c). Expression of EIF4A-regulated C-MYC was also reduced by knocking down ZNF143 (Fig.7d).

Next, we performed chromatin immunoprecipitation (ChIP) assay to confirm the direct binding of ZNF143 to the EIF4A2 promoter (Fig. 7e). Moreover, dual-luciferase assays indicated a significant increase in luciferase activities after co-transfection of ZNF143 plasmid and the wild-type EIF4A2 promotor plasmid, but not with the mutant-type EIF4A2 promotor plasmid (Fig. 7f), which verified EIF4A2 as a transcriptional target of ZNF143. In conclusion, EIF4A2 promotes experimental metastasis and oxaliplatin resistance in CRC. Silvestrol inhibits tumor growth, invasion, migration, cancer stemness and improves oxaliplatin resistance (Fig. 7g).

## Discussion

Dysregulation of translation can be considered as a hallmark of cancer and is associated with caner metastasis, changes in immune response and cancer metabolism [34, 35]. At present, most of the studies on cancer translation dysregulation focus on translation initiation [36–38]. EIF4A2 is an important family member of EIF4A and EIF4A2 had been reported to be critical for miRNA-mediated gene silencing [12]. Complete elimination of EIF4A2 has been published to not be essential for cell survival in NIH/3 T3 and HELA cell [21]. Moreover, Large-Scale, Deep RNAi Screening in Project DRIVE across 398 cancer cell lines also indicates that EIF4A2 is not essential for cell proliferation [39]. However, little attention was paid to the effects of EIF4A2 in experimental metastasis and oxaliplatin resistance in CRC.

In our study, expression of EIF4A2 was elevated in CRC patients and associated with poorer survival. High EIF4A2 was also associated with more distant metastasis. After PSM, high EIF4A2 expression was still an independent prognostic factor of shorter OS in 81 pairs of CRC patients, which made our conclusions more convincing. High EIF4A2 was also associated with shorter OS, TTP and lower response rate in 74 CRC patients who received first-line oxaliplatin-based chemotherapy regimen. All these results suggest that abnormal EIF4A2 expression may be associated with tumor metastasis and oxaliplatin resistance in CRC.

High EIF4A2 level was prognostic of poor prognosis in liver cancer, head and neck cancer, melanoma and prostate cancer by TCGA data analysis, which was in accordance with our results that EIF4A2 was associated with poor prognosis in CRC. High EIF4A2 expression in non-small cell lung cancer and breast cancer was reported to be associated with better prognosis [15, 16], which contradicted with our findings here. One explanation may be due to the different tumor microenvironment and resulting complicated regulatory pathway networks in tumors.

To clarify the mechanisms of EIF4A2 promoting metastasis and oxaliplatin resistance, we conducted cellular experiments, mouse experiments and tested the therapeutic roles of silvestrol in the CDX and PDX models. To our knowledge, this is the first study to systematically evaluate the role of EIF4A2 in experimental metastasis and oxaliplatin resistance. Our results showed that EIF4A2 acted as an oncogene in CRC. Genetic knockdown of EIF4A2 inhibited cell migration and invasion in vitro as well as lung metastasis in vivo. Inhibition of EIF4A2 also improved oxaliplatin sensitivity and had synergistic effects with oxaliplatin in mouse models.

The CRC patients with early-stage disease are often treated with curative surgery in combination with adjuvant chemotherapy regimens such as FOLFOX or XELOX that includes oxaliplatin. Nevertheless, many CRC patients still relapse and have distant metastasis after receiving multiple treatments. For metastatic CRC patients at first diagnosis, oxaliplatin is one of the backbone drugs that could prolong OS but many patients would eventually develop oxaliplatin resistance. Our study implied that pharmacological inhibition of EIF4A by silvestrol may be an effective therapeutic strategy for overcoming oxaliplatin resistance. Silvestrol targets at EIF4A1 and EIF4A2, silvestrol also have the potential to act as cancer immunotherapies in melanoma [40]. Pharmacological inhibition of EIF4A by sivestrol may play powerful anti-cancer immune effects by downregulation of PDL1 in cancer cells. FOLFOX was reported to induce PD-L1 expression and high CD8 T cell infiltration in the tumor microenvironment of CRC patients [41]. Synergistic effects between oxaliplatin and silvestrol confirmed by the PDX experiment are encouraging. Combination of oxaliplatin and silvestrol may be worth of further clinical trials. However, major disadvantages of silvestrol include poor bioavailability coupled with high potential to develop multidrug resistance [37]. Further efforts on modifying molecular structures of silvestrol should be made to overcome these disadvantages.

Moreover, we found that EIF4A2 could affect the stemness of CRC cells. Our results are in accordance with previous studies, which indicated that cancer stem cells were responsible for cancer invasiveness and metastasis [42]. We also showed that *C-MYC* mRNA and protein significantly reduced after knockdown of *EIF4A2*. It has been reported that the most EIF4A-dependent and silvestrol-sensitive transcripts are oncogenes, super-enhancer-associated transcription factors, and epigenetic regulators, such as NOTCH1, MYC, MYB and ETS1 [37]. Among these oncoproteins, MYC deregulation occurred in more than half of human cancers and is usually correlated with aggressive phenotypes, drug resistance and unfavorable prognosis. MYC is also thought to be very important in cancer cell apoptosis and differentiation [43]. In addition, MYC family members play crucial roles in stem cell biology, and MYC-dependent metabolic reprogramming is tightly related to regulation of CD44-variant-dependent redox-stress in cancer stem cells [43]. MYC has long been considered as a promising target for cancer treatment. However, directly targeting MYC seems to be an impossible mission due to its undruggable protein structure [44]. Thus, targeting MYC translation might be an alternative way. Based on our results, it is conceivable that targeting EIF4A2-mediated translation is a promising strategy for MYC inhibition.

Next, we further explored on upstream regulation of *EIF4A2 and* found that ZNF143 might be a specific transcription factor for *EIF4A2*. Firstly, we found a strong positive correlation between *ZNF143* and *EIF4A2* in CRC tissues. Secondly, knocking-down *ZNF143* significantly reduced *EIF4A2*. Thirdly, ChIP assays stringently confirmed ZNF143 could bind to the *EIF4A2* promoter. Moreover, binding of ZNF143 to *EIF4A2* showed specific transcription activity validated by co-transfection of luciferase vector driven by wild-type or mutated *EIF4A2* promoter. About 2000 gene promoters contain the binding sites of ZNF143, and these genes play important roles in cell cycle progression, cell invasion and migration [45].

The mechanism of how EIF4A2 affects translation in CRC requires further investigation. Therefore, we plan to perform exquisite Ribosome-sequencing to compare the complete translation differences between cells with knocking-down EIF4A2 and control cells in the future. EIF4A is the enzyme core of the EIF4F complex, and EIF4A operates continuous synthesis of protein when it is associated with EIF4G, EIF4B, and EIF4H. EIF4A1 and EIF4A2 share more than 90% homologous sequences, both of which are involved in protein translation, and seem to be functionally interchangeable. However, several studies suggest there are delicate differences in the functions of EIF4A1 and EIF4A2. EIF4A2 increases when EIF4A1 is inhibited, but EIF4A2 could not compensate for all the functions of EIF4A1. There are also tissue differences in EIF4A1 and EIF4A2 expression, but the total mole concentration of EIF4A appears to be constant across all cell types. EIF4A is most abundant of all translation initiation factors, with 3 copies per ribosome, yet under certain circumstances EIF4A is still not enough [46]. In addition, EIF4A is negatively regulated by PDCD4, both on the expression and activity levels. PDCD4 depolymerizes EIF4A from EIF4G and RNA and thus inhibits mRNA translation. The interaction between EIF4A and PDCD4 is regulated by mTORC1, adding complexity of the control network of EIF4A2 [47].

## Conclusion

In this study, high EIF4A2 expression predicts poor prognosis of CRC patients and is associated with distant metastasis and poor response to oxaliplatin. Knocking-down EIF4A2 inhibits sphere formation and experimental metastasis, as well as oxaliplatin resistance in CRC. The effects of EIF4A2 may be conducted by its translation target c-Myc. EIF4A inhibitor silvestrol has synergistic effects with oxaliplatin and dramatically inhibits tumor growth in CDX and PDX models. Our study suggests that silvestrol in combination with oxaliplatin may represent a novel therapeutic strategy for treating metastatic CRC patients.

## Additional file

Additional file 1: **Figure S1.** Prognostic value of EIF4A1, EIF4A2 and EIF4A3. **Figure S2.** A prognostic nomogram based on EIF4A2. **Figure S3.** EIF4A2 expression profile in CRC cell lines. **Figure S4.** EIF4A2 overexpression in the DLD1 and HCT116 cell lines with EIF4A2 stably knocked down. **Figure S5.** Effects of silvestrol on cell invasion, metastasis and apoptosis. **Figure S6.** ZNF143 mRNA level in tumor samples and normal tissues. **Table S1.** Sequences of siRNA. **Table S2.** Primers for qRT-PCR. **Table S3.** Univariate and multivariate analyses of prognostic factors for DFS of 245 CRC patients under curative surgery. **Table S4.** Univariate and multivariate analyses of prognostic factors for PFS of 52 metastatic CRC patients. **Table S5.** Comparison of demographic and clinical characteristics of 162 patients with colorectal cancer after PSM. **Table S6.** Univariate and multivariate analyses of prognostic factors for OS of 162 CRC patients after PSM. **Table S7.** Gene lists of Metastasis array. (DOCX 4314 kb)

## Abbreviations

AJCC: American Joint Committee on Cancer
CDX: Cell-derived xenograft
ChIP: Chromatin immunoprecipitation
CRC: Colorectal cancer
DFS: Disease free survival
dMMR: Deficient mismatch repair
EIF4A2: Eukaryotic initiation factor 4A2
IC50: The half-maximal inhibitory concentration (IC50)
IHC: Immunohistochemistry
OS: Overall survival
PDX: Patient-derived xenograft
PFS: Progression free survival
PSM: Propensity score matching
qRT-PCR: Quantitative realtime polymerase chain reaction; sh, short hairpin
ROC: Receiver operating characteristics curve
TCGA: The Cancer Genome Atlas
TNM: Tumor-node-metastasis
TUNEL: TdT-mediated dUTP Nicked-end Labeling
