# Supplementary material for: Eukaryotic initiation factor 4A2 promotes experimental metastasis and oxaliplatin resistance in colorectal cancer
Source: J Exp Clin Cancer Res. 2019 May 14;38:196. doi: 10.1186/s13046-019-1178-z (PMC6518650; doi:10.1186/s13046-019-1178-z)
Supplement: Supplementary file 1 — Figure S1. Prognostic value of EIF4A1, EIF4A2 and EIF4A3. Figure S2. A prognostic nomogram based on EIF4A2. Figure S3. EIF4A2 expression profile in CRC cell lines. Figure S4. EIF4A2 overexpression in the DLD1 and HCT116 cell lines with EIF4A2 stably knocked down. Figure S5. Effects of silvestrol on cell invasion, metastasis and apoptosis. Figure S6. ZNF143 mRNA level in tumor samples and normal tissues. Table S1. Sequences of siRNA. Table S2. Primers for qRT-PCR. Table S3. Univariate and multivariate analyses of prognostic factors for DFS of 245 CRC patients under curative surgery. Table S4. Univariate and multivariate analyses of prognostic factors for PFS of 52 metastatic CRC patients. Table S5. Comparison of demographic and clinical characteristics of 162 patients with colorectal cancer after PSM. Table S6. Univariate and multivariate analyses of prognostic factors for OS of 162 CRC patients after PSM. Table S7. Gene lists of Metastasis array. (DOCX 4314 kb) [file 13046_2019_1178_MOESM1_ESM.docx]

**Supplementary figures and tables**


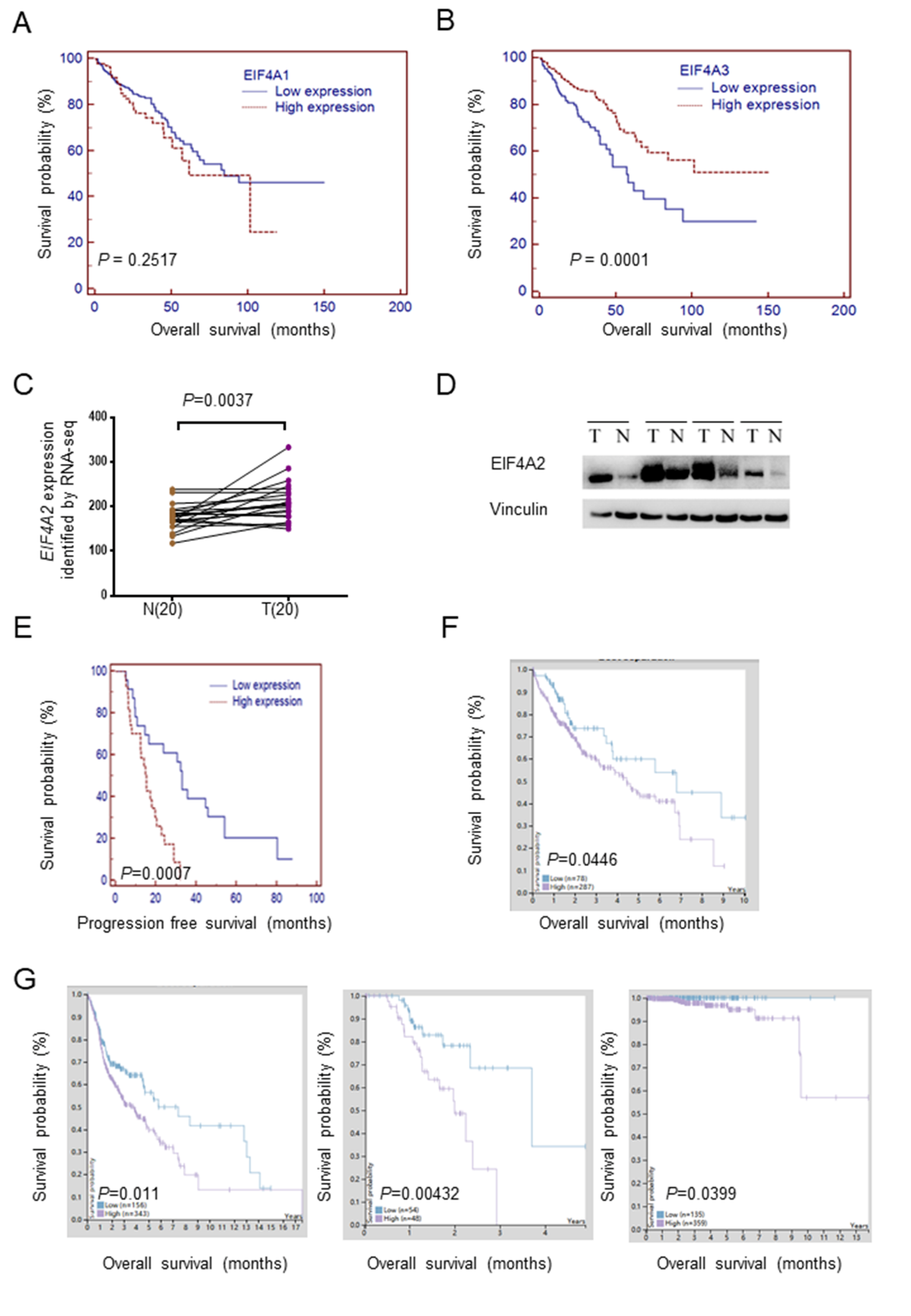


Figure s1. Prognostic value of EIF4A1, EIF4A2 and EIF4A3

(A, B) *EIF4A1* was not associated with prognosis while high level of *EIF4A3* was significantly associated with better prognosis of CRC patients in TGCA data analysis.

(C) RNA sequencing data of 20 paired adjacent normal and colorectal cancer samples showed that *EIF4A2* significantly increased in tumors.

(D) Western blot analysis showed that EIF4A2 in tumors was higher in 4 paired normal and CRC samples.

(E) The progression-free survival curve of 52 metastatic CRC patients with low and high expression of EIF4A2 were generated using the Kaplan-Meier method (log-rank test).

(F, G) High level of EIF4A2 was prognostic of poor prognosis in liver cancer, head and neck cancer, melanoma and prostate cancer in TCGA patient data analysis.


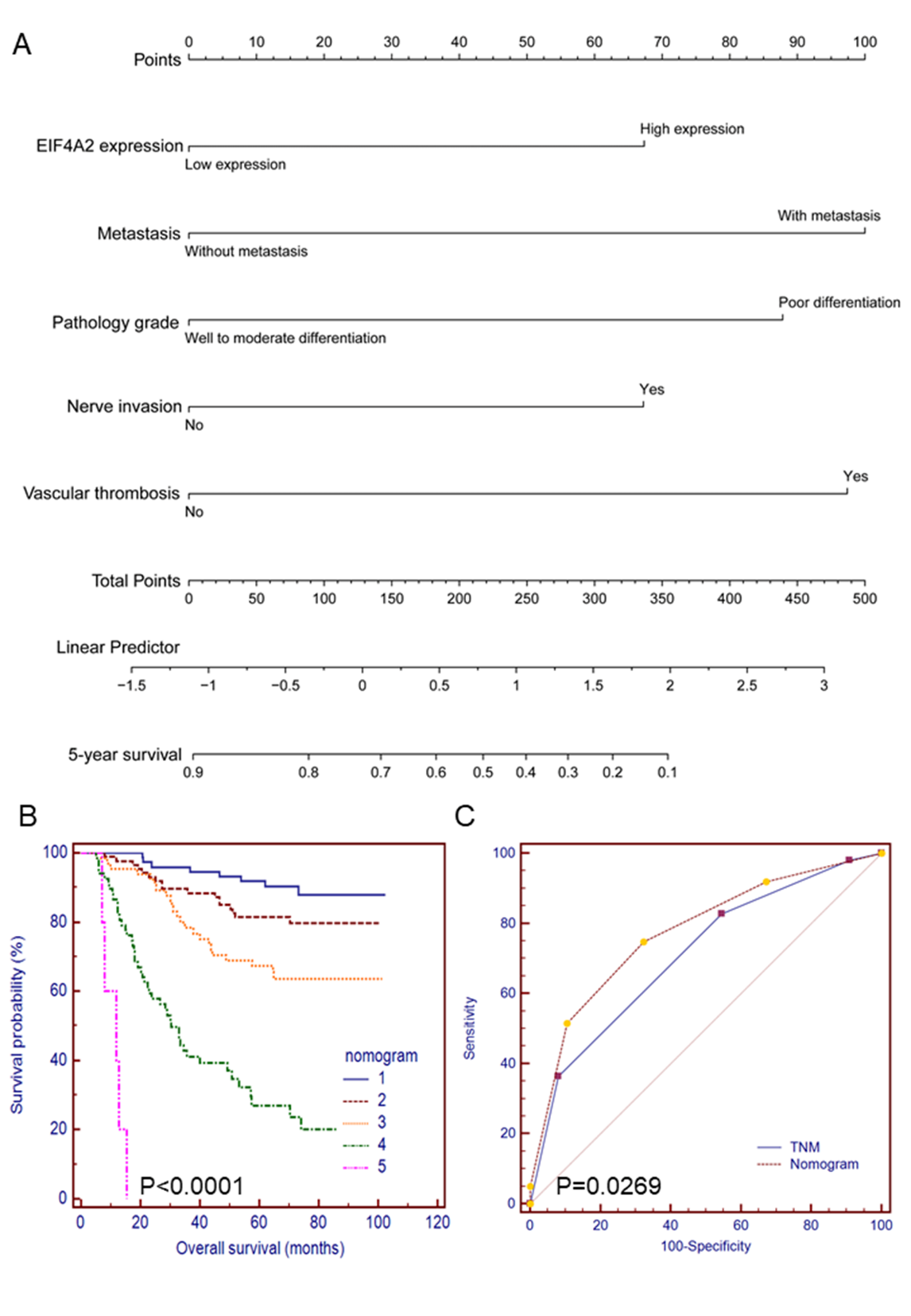


Figure s2. A prognostic nomogram based on EIF4A2.

(A) A prognostic nomogram including EIF4A2, distant metastasis, pathology grade, nerve invasion and vascular thrombosis was established.

(B) Kaplan-Meier curves analysis of the nomogram.

(C) The AUC (area under curves) of the Nomogram was significantly larger than that of the TNM staging system (0.773 vs 0.710, *P*=0.0269).


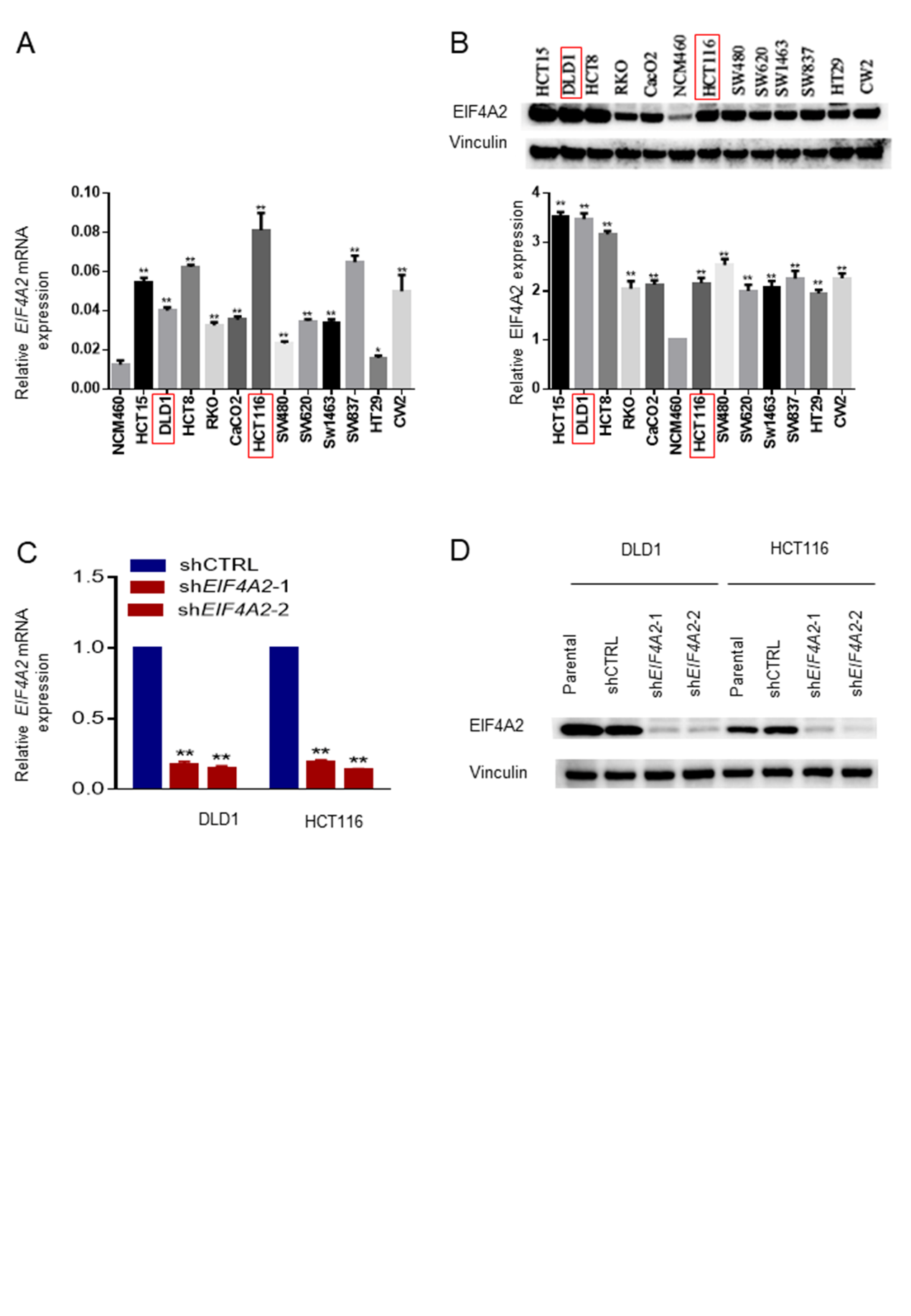


Figure s3. EIF4A2 expression profile in CRC cell lines

(A, B) RT-qPCR (A) and immuno-blot analysis (B) showed high expression of EIF4A2 compared to the normal human colon epithelial cell line NCM460.

(C, D) The indicated cells were transected with control shRNA or shRNAs targeting *EIF4A2*. The efficiency of *EIF4A2* knockdown was shown by RT-qPCR (C) and immune-blot analysis.

*, P < 0.05; **, P < 0.01 versus the control


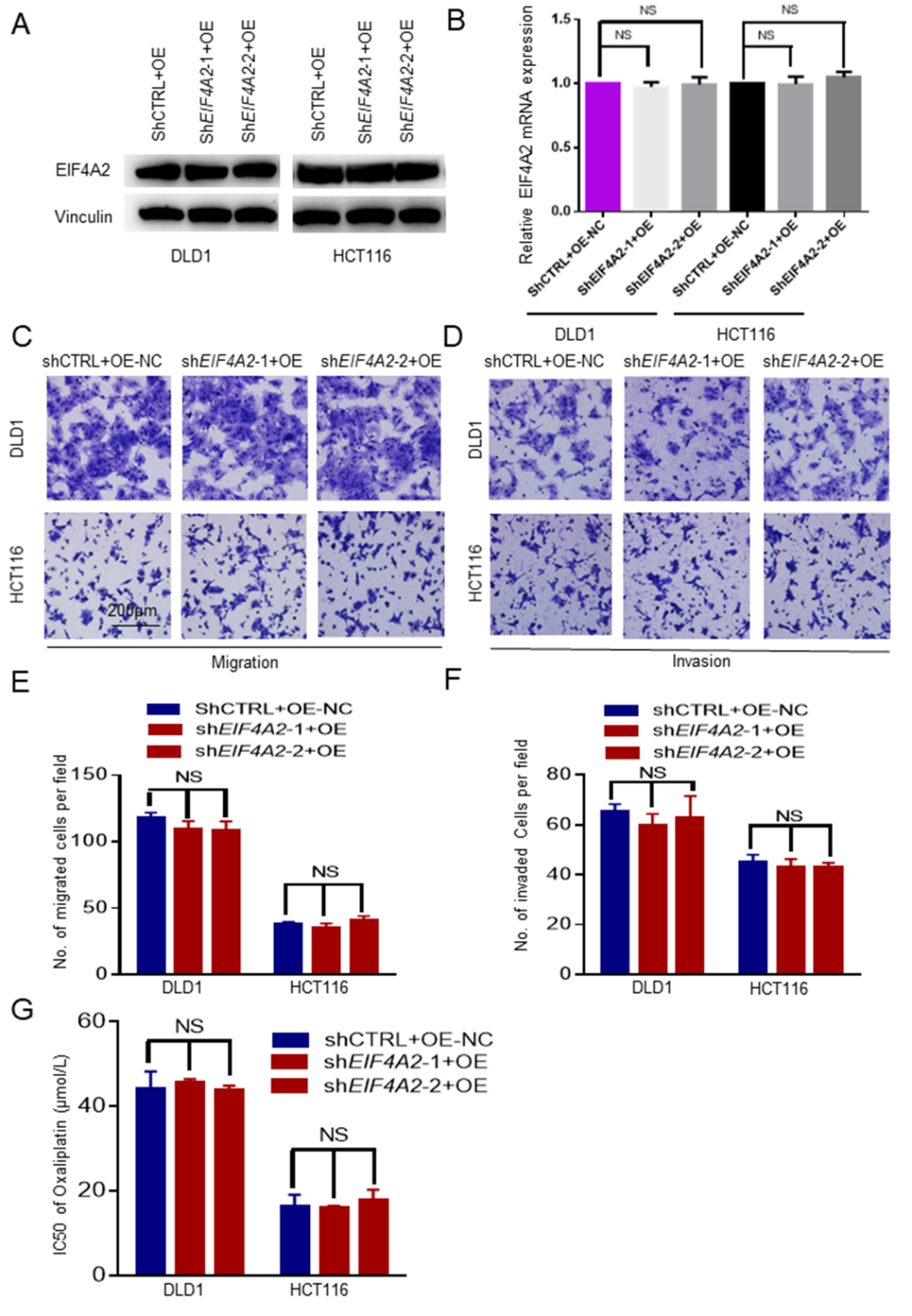


Figure s4. EIF4A2 overexpression in the DLD1 and HCT116 cell lines with EIF4A2 stably knocked down.

(A, B) immuno-blot analysis (A) and RT-qPCR (B) and showed expression of EIF4A2 recovered when we overexpressed EIF4A2 in the DLD1 and HCT116 cell lines with EIF4A2 stably knocked down.

(C, D, E, F, G) The migration ability, invasion ability and IC50 of oxaliplatin of sh-EIF4A2-1+OE and sh-EIF4A2-2+OE in DLD1 and HCT116 cell lines recovered as well as the control group. *, P < 0.05; **, P < 0.01 versus the control


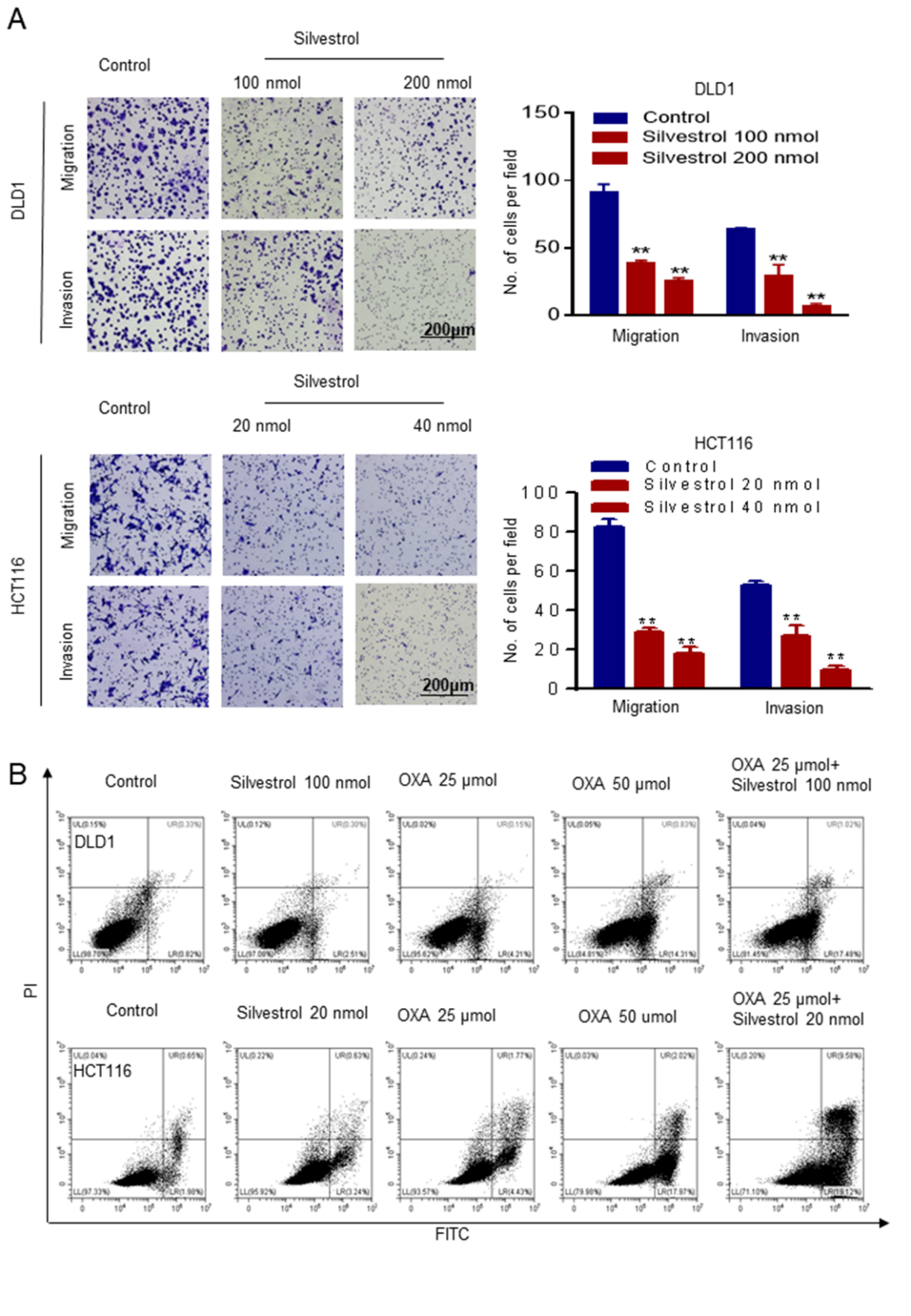


Figure s5 Effects of silvestrol on cell invasion, metastasis and apoptosis

(A) Silvestrol significantly inhibited transwell-migration and invasion of HCT116 and DLD1 cells in a concentration-dependent manner.

(B) Representative images of cell apoptosis of the indicated cells treated with silvestrol in Annexin-V/propidium iodide (PI) assays. *, P < 0.05; **, P < 0.01 versus the control


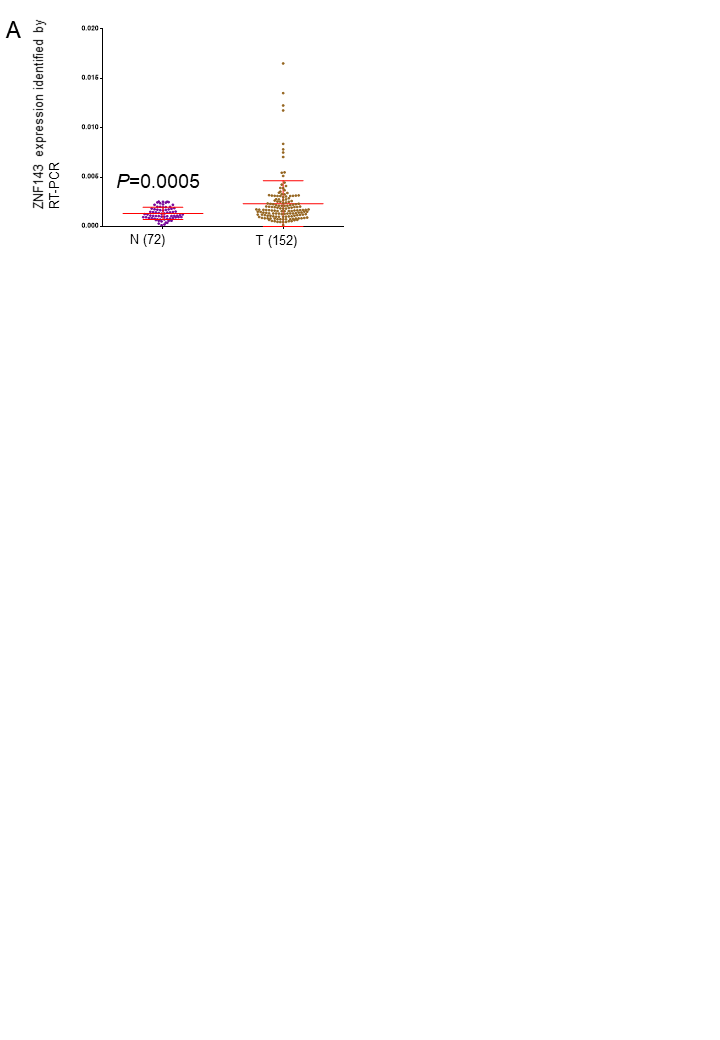


Figure s6 *ZNF143* mRNA level in tumor samples and normal tissues

(A) Level of *ZNF143* mRNA was significantly higher in 152 tumor samples than in 72 normal tissues.

Supplementary table 1: sequences of siRNA

| **SiRNA name** | **Sense** | **Anti-sense** |
| --- | --- | --- |
| siETV4-1 | GCGUUGUCCCUGAGAAAUUTT | AAUUUCUCAGGGACAACGCTT |
| siETV4-2 | GCUGGAUGACCCAACAAAUTT | AUUUGUUGGGUCAUCCAGCTT |
| siETV4-3 | CCCUCUUCUCUUUGGCCUUTT | AAGGCCAAAGAGAAGAGGGTT |
| siE2F6-1 | GACUUAAACAAGGUUGCAATT | UUGCAACCUUGUUUAAGUCTT |
| siE2F6-2 | GAAGCUACAGGAGGAACUUTT | AAGUUCCUCCUGUAGCUUCTT |
| siE2F6-3 | CCAUGAACAGAUCGUCAUUTT | AAUGACGAUCUGUUCAUGGTT |
| siZBTB33-1 | CCACCAAAUGUCAGUUCUUTT | AAGAACUGACAUUUGGUGGTT |
| siZBTB33-2 | GCAAAUAUCGGUGAAGAUATT | UAUCUUCACCGAUAUUUGCTT |
| siZBTB33-3 | GCCAAUGACCUGGGAAGAUTT | AUCUUCCCAGGUCAUUGGCTT |
| siATF4-1 | CUCCCAGAAAGUUUAACAATT | UUGUUAAACUUUCUGGGAGTT |
| siATF4-2 | CUGCUUACGUUGCCAUGAUTT | AUCAUGGCAACGUAAGCAGTT |
| siATF4-3 | GUGAGAAACUGGAUAAGAATT | UUCUUAUCCAGUUUCUCACTT |
| Negative Control | UUCUCCGAACGUGUCACGUTT | ACGUGACACGUUCGGAGAATT |

Supplementary table 2: primers for qRT-PCR

| **Gene name** | **Forward** | **Reverse** |
| --- | --- | --- |
| EIF4A2 | TGGTGTCATCGAGAGCAACTG | GGCTTCTCAAAACCGTAAGCA |
| β-Actin | TGGATCAGCAAGCAGGAGTA | TCGGCCACATTGTGAACTTT |
| ZNF143 | GTACAGGGGACAGTTTGCGTC | TGGAGGTGTGGTGAATAAATGC |
| EIF4A1 | AAGGCGTCATCGAGAGTAACT | ATGTGGCCGTTTTCCCAGTC |
| METAP2 | AAAGGACAAGAATGCGAATACCC | CAGGCTTGATCCAGCTCATTAC |
| RPL13A | GCCATCGTGGCTAAACAGGTA | GTTGGTGTTCATCCGCTTGC |
| RB1 | CTCTCGTCAGGCTTGAGTTTG | GACATCTCATCTAGGTCAACTGC |
| PTEN | TGGATTCGACTTAGACTTGACCT | GGTGGGTTATGGTCTTCAAAAGG |
| RPSA | GTGGCACCAATCTTGACTTCC | GCAGGGTTTTCAATGGCAACAA |
| DENR | ACAGTGCCAAGTTAGATGCCG | TCCTTGACCCTCACTAATTCCA |
| CD44 | CTGCCGCTTTGCAGGTGTA | CATTGTGGGCAAGGTGCTATT |
| TNFSF10 | TGCGTGCTGATCGTGATCTTC | GCTCGTTGGTAAAGTACACGTA |
| GNRH1 | CAAAAACTCCTAGCTGGCCTT | CAGTTGACCAACCTCTTTGACT |
| HTATIP2 | CGGAGGGATTTGTTCGTGTTG | AGCTCCTTTAGAGGATAGCAAGT |
| HPRT1 | CCTGGCGTCGTGATTAGTGAT | AGACGTTCAGTCCTGTCCATAA |
| IL18 | TCTTCATTGACCAAGGAAATCGG | TCCGGGGTGCATTATCTCTAC |
| SMAD4 | ACGAACGAGTTGTATCACCTGG | TGCACGATTACTTGGTGGATG |
| NME1 | AAGGAGATCGGCTTGTGGTTT | CTGAGCACAGCTCGTGTAATC |
| MDM2 | GAATCATCGGACTCAGGTACATC | TCTGTCTCACTAATTGCTCTCCT |
| NME2 | CCACCTCTTATTCATAGACCCA | AGATTCAAAGCCAGGCACCAT |
| SET | AGCAAGAAGCGATTGAACACA | TGGTTGGCGGAGTTTGTTATATT |
| KRAS | ACAGAGAGTGGAGGATGCTTT | TTTCACACAGCCAGGAGTCTT |
| CCL7 | CAAGACCAAACTGGACAAGGAGAT | AGAACCACTCTGAGAAAGGACAGG |
| SMAD2 | CGTCCATCTTGCCATTCACG | CTCAAGCTCATCTAATCGTCCTG |
| IL1B | ATGATGGCTTATTACAGTGGCAA | GTCGGAGATTCGTAGCTGGA |
| B2M | GAGGCTATCCAGCGTACTCCA | CGGCAGGCATACTCATCTTTT |
| ETV4 | GATGAAAGCCGGATACTTGGAC | TTCGCGCAAGCTCCCATTT |
| CXCR4 | ACTACACCGAGGAAATGGGCT | CCCACAATGCCAGTTAAGAAGA |
| CTSK | ACACCCACTGGGAGCTATG | GACAGGGGTACTTTGAGTCCA |
| MTSS1 | CAGTCCCAGCTTCGGACAAC | TGAGAGCAGATCCAATCTCCC |
| PNN | GTCGCCGTGAGAACTTTGC | GGTCCTCCTCCACTATCTGAGA |
| CTSL1 | CTTTTGCCTGGGAATTGCCTC | CATCGCCTTCCACTTGGTC |
| VEGFA | AGGGCAGAATCATCACGAAGT | AGGGTCTCGATTGGATGGCA |
| TIMP4 | CCACTCGGCACTTGTGATTC | CATCCTTGACTTTCTCAAACCCT |
| MET | AGCAATGGGGAGTGTAAAGAGG | CCCAGTCTTGTACTCAGCAAC |
| MMP7 | GAAAGAAATAGAAACTTCAGGCAGA | GAGTGGAGGAACAGTGCTTATC |
| TRPM1 | CAGACAGTAAGTTTTCCATCCC | GAGTACAGTTCAATCACGGACC |
| MMP10 | CAGTAGACAAAGAAGGTAAGGG | AGAGGATAGGCAGAGCAGA |
| MYCL1 | GATGGATGGAGATGTGGAAAT | CGACTCGGAGAATGAAGAAAT |
| MMP3 | GGAGACTTTTACCCTTTTGATGG | TGGTCCCTGTTGTATCCTTTGT |
| CDKN2A | CCCCGATTGAAAGAACCAGAGAG | TACGGTAGTGGGGGAAGGCATA |
| RORB | GCTTGATTTCAGTGCTTATTGTGTC | TGGGTCTTCTCTTTCTACCTTTTCT |
| HPSE | TCTTCCTTGGTAGCAGTCCGT | TTTCATCAATGGGTCGCAGTT |
| CXCR2 | CCCTGCCTGTCTTACTTTTCCGA | ATCCGCCAGTTTGCTGTATTGTTG |
| APC | CTTCCTCTCCTCATCCAGCTTTTAC | ACGCCTGCCTCTCTTGTCATC |
| SSTR2 | GGACCACCACAAAGTCAAACA | GCTTCCCTTCTACATATTCAACG |
| HGF | CTCTGGTTCCCCTTCAATAGCAT | TTCCCTTGTAGCTGCGTCCT |
| FXYD5 | GTTTCATCAGCAGGCCAGGTT | ACATTCAGGTCCCGACACGAG |
| CDH11 | CAGATAAAGCAATCTCATGTCTTCC | GTAGCACCAACACCCTCACCA |
| KISS1 | CCTGCCGAACTACAACTGGAAC | TCCCTTAGCCCTACGTCCC |
| MYC | CGTCCTCGGATTCTCTGCTC | CGATTTCTTCCTCATCTTCTTGTTC |
| MMP13 | ATACTACCATCCTACAAATCTCG | CATCTAAGGTGTTATCGTCAAGT |
| MGAT5 | GGGAACCCAAGTCCAACAAAC | TGAAATCAAAAGGCAGAACCAG |
| SYK | CTTCACTTCCTTTCATCCCTC | CTAGTTACCCAACATTACGCC |
| KISS1R | CCGAGACCTGCTGGATGTAGT | ACGTGACCTTCCTCCTGTGCT |
| CDH1 | CAAATCCAACAAAGACAAAGAAGGC | ACACAGCGTGAGAGAAGAGAGT |
| IGF1 | TGTCCTCCTCGCATCTCTTCTACC | CCCCTGTCTCCACACACGAACT |
| NR4A3 | CAGTGGGACAGTATCTGGAATAA | GTCTCAGTGTTGGAATGGTAAA |
| CDH6 | TGGACAACAAATGTACCGACA | AGCTCAAGCTATAAACAGAAGGAC |
| CXCL12 | TCACATCTAACCTCATCTTCTTCAC | ACTCTTCACATAGCACATTGTTCTC |
| CST7 | TCAGTGACAACGGAGAACAGG | GGAGGTGGAAATTGGCAGAAC |
| TP53 | AGCTTTGAGGTGCGTGTTTGTG | TCTCCATCCAGTGGTTTCTTCTTTG |
| TIMP2 | GCTTTCATTCGTCTCCCGTCTTT | CGGCTCTTCTTAACCTGTTTTGTTT |
| MTA1 | TTGTCTGTGAGTGGGTTGTGC | TGTTAAAAGAAGGCGAGGAGG |
| ITGB3 | TTACCTCCTAATTCCACACCCTCAC | CTGGCTCTACAATAGCACTCTCTCC |
| ITGA7 | CCTTGAACTGCTGTCGGTCTT | ACTTGATGCTCCGAGATGCCT |
| TSHR | CTGGAATCACACTCCTTCTACA | TGGAATAAACTTTGGTCAGGTC |
| FN1 | AAGCCCATAGCTGAGAAGTGTTTTG | GGATGTCCTTGTGTCCTGATCGT |
| NME4 | TGATGTGGACGCTGAAGTCAC | AGGTCTGGGAAGGGTACAATG |
| GAPDH | GGACCTGACCTGCCGTCTAG | GTAGCCCAGGATGCCCTTGA |
| CTNNA1 | CGTCGCCTCTACCAAATACC | CTTCTGAGATGCCCGTTTA |
| TIMP3 | CTATCGGTATCACCTGGGTTGTA | ATGCAGGCGTAGTGTTTGGA |
| MMP9 | CCACCCTTGTGCTCTTCCCTG | TCTGCCACCCGAGTGTAACCA |
| FGFR4 | ACGAGACTCCAGTGCTGATGG | TCGAATAGGCACAGTTACCCC |
| MMP11 | GGTCTTGGTAGGTGCCTGCATC | CCTCCCCATTTGACTGTGAACTTT |
| PLAUR | TGGCCGGGCTGTCACCTATT | TTGGACGCCCTTCTTCACCTT |
| FAT1 | TTCTCACCAGTGCCTTTTGTT | TTGAATCCATCCACCCTCCTA |
| MCAM | TGAGGACTGGCAGTGGAAGTG | CGGCAAGTGAACAAGACCAAG |
| TGFB1 | GAAACCCACAACGAAATCTATGAC | ACGTGCTGCTCCACTTTTAACT |
| MMP2 | AACTACAACTTCTTCCCTCGCAA | CAAAGGCATCATCCACTGTCTCT |
| FLT4 | GATGGTGGTCACATAGAAGTAGAT | TGGAGGGAAAGAATAAGACTGT |
| CD82 | TTCAGTCAGGATGGGCAAGAG | CCATTCCGAAGACTACAGCAA |
| EPHB2 | AACGTGTTTGAGTCAAGCCAGAA | ACGCACCGAAAACTTCATCTCC |
| TCF20 | CGGGTAATGGTATCGGAAGGA | GGTTTGTGGCAGGCTCTATGG |
| COL4A2 | CAACAGAGGACTTGGTTTCTACGGA | TGTACTGATCTGGGTGGAAGGTGA |
| BRMS1 | GCAGTTTGTCATCCCACCATT | GGAGCCTCAAGATTCGCATTC |
| SRC | ATCACTTCCTTGCCCCCATTTC | CATCCTCAGACCCCTTGTTTCCT |
| NF2 | TGTATCGGGAACCATGATCTATTTA | CTCCATCTGCTTTCTAGCCTTCT |
| CHD4 | AAGTCTTCTTGGTAACTGTGGC | GATCTGACCCCTATTGTGGTAG |
| ACTB | CTACCTCATGAAGATCCTCACCGA | TTCTCCTTAATGTCACGCACGATT |
| HRAS | GTGGAATCTCGGCAGGCTCA | CGCACCAACGTGTAGAAGGCAT |
| EWSR1 | CTGGTAGGAGGGTAGGATGGA | TGGAAACAAGCCCACTGAGAC |
| CTBP1 | AAAGCTGAAGGGTTCCGACTC | CTCAACGAGCACAACCACCAC |
| CD24 | TGAAGAACATGTGAGAGGTTTGAC | GAAAACTGAATCTCCATTCCACAA |
| CD105 | CGCCAACCACAACATGCAG | GCTCCACGAAGGATGCCAC |
| CD133 | TGGATGCAGAACTTGACAACGT | ATACCTGCTACGACAGTCGTGGT |
| ABCC2 | ATGCAGCCTCCATAACCATGA | CTTCGTCTTCCTTCAGGCTATTCA |
| ABCG2 | TCATCAGCCTCGATATTCCATCT | GGCCCGTGGAACATAAGTCTT |
| Notch | CCTGAGGGCTTCAAAGTGTC | CGGAACTTCTTGGTCTCCAG |
| Nanog | AATACCTCAGCCTCCAGCAGATG | TGCGTCACACCATTGCTATTCTTC |
| OCT4 | CTTGCTGCAGAAGTGGGTGGAGGAA | CTGCAGTGTGGGTTTCGGGCA |
| Bmi1 | TGGAGAAGGAATGGTCCACTTC | GTGAGGAAACTGTGGATGAGGA |
| Smo | TGGTCACTCCCCTTTGTCCTCAC | GCACGGTATCGGTAGTTCTTGTAGC |
| Aldh1 | TTGGAATTTCCCGTTGGTTA | CTGTAGGCCCATAACCAGGA |
| EIF4A2 promotor 1 | GAGACAACAAGGATGTCTGACG | AACCCTACCCATGGTGCAAAATA |
| EIF4A2 promotor 2 | TGAGTCAGCTTTCCCAGTAATGAT | TGGACGCTTTGGCAGATACA |
| EIF4A2 promotor 3 | ACCGATGTAGTCCCAAAGCG | TAGGGAAAATGTCGGCCACC |
| EIF4A2 promotor 4 | GGTGGCCGACATTTTCCCTA | GGGTTGGGTTGGGACTCTTT |
| EIF4A2 promotor 5 | GCGGGGAAAGCGAGGTTTA | GCCACCAGACATGATCCGAA |
| EIF4A2 promotor 6 | CAGGTATGCAGTCTGTTGGC | GTGTACCGAACTCGGACTGC |

Supplementary table 3: Univariate and multivariate analyses of prognostic factors for DFS of 245 CRC patients under curative surgery

| Variable | Univariate | | Multivariate | | | | |
| --- | --- | --- | --- | --- | --- | --- | --- |
|  | Log-rank χ^2^ | P value | B | SE | HR | 95% CI | *P* value |
| Gender (male/female) | 2.205 | 0. 138 |  |  |  |  |  |
| Age, years (> 57/≤ 57) | 0.911 | 0.340 |  |  |  |  |  |
| Lymph node metastasis (Yes/No) | 10.828 | 0.001* | -0.526 | 0.744 | 0.591 | 0.139 to 2.521 | 0.480 |
| Vascular thrombosis (Yes/No) | 42.319 | <0.001* | 0.927 | 0.259 | 2.527 | 1.524 to 4.190 | <0.001 |
| Pathology differentiation (Well and moderately differentiated/poor differentiation) | 2.800 | 0.094 |  |  |  |  |  |
| TNM (AJCC 7th)  (Ⅰ-Ⅱ/ Ⅲ) | 13.330 | <0.001* | 1.010 | 0.786 | 2.746 | 0.593 to 12.713 | 0.199 |
| Invasion depth (Whole layer/ no whole layer) | 1.282 | 0.258 |  |  |  |  |  |
| Nerve invasion (yes /no) | 31.583 | <0.001* | 0.833 | 0.235 | 2.300 | 1.454 to 3.640 | <0.001 |
| EIF4A2(high expression/low expression) | 4.700 | 0.030* | 0.438 | 0.219 | 1.550 | 1.011 to 2.374 | 0.045 |

Supplementary table 4: Univariate and multivariate analyses of prognostic factors for PFS of 52 metastatic CRC patients

| Variable | Univariate | | Multivariate | | | | |
| --- | --- | --- | --- | --- | --- | --- | --- |
|  | Log-rank χ^2^ | P value | B | SE | HR | 95% CI | *P* value |
| Gender (male/female) | 3.508 | 0.061 |  |  |  |  |  |
| Age, years (> 57/≤ 57) | 2.129 | 0.145 |  |  |  |  |  |
| Lymph node metastasis (Yes/No) | 2.368 | 0.124 |  |  |  |  |  |
| Pathology differentiation (Well and moderately differentiated/poor differentiation) | 8.120 | 0.004* | 1.098 | 0.422 | 2.997 | 1.316 to 6.829 | 0.009 |
| Nerve invasion (yes /no) | 0.811 | 0.368 |  |  |  |  |  |
| EIF4A2(high expression/low expression) | 11.422 | 0.001* | 1.286 | 0.409 | 3.618 | 1.630 to 8.030 | 0.002 |

Supplementary table 5: Comparison of demographic and clinical characteristics of 162 patients with colorectal cancer after PSM

| Variable | low EIF4A2  n (%) | high EIF4A2  n (%) | *P* value |
| --- | --- | --- | --- |
| Total | 81(50.0) | 81(50.0) |  |
| Age, years |  |  | 1.000 |
| >57 | 38 (46.9%) | 38 (46.9%) |  |
| ≤57 | 43 (53.1%) | 43 (53.1%) |  |
| Gender |  |  | 1.000 |
| male | 51 (63.0%) | 51 (63.0%) |  |
| female | 30 (37.0%) | 30 (37.0%) |  |
| Lymph node metastasis |  |  | 1.000 |
| N0-1 | 63 (77.8%) | 63 (77.8%) |  |
| N2 | 18 (22.2%) | 18 (22.2%) |  |
| Nerve invasion |  |  | 1.000 |
| No | 39 (48.2%) | 39 (48.2%) |  |
| Yes | 42 (51.8%) | 42 (51.8%) |  |
| Invasion depth |  |  | 1.000 |
| No whole layer | 4 (4.94%) | 4 (4.94%) |  |
| Whole layer | 77 (95.1%) | 77 (95.1%) |  |
| Vascular thrombus |  |  | 1.000 |
| 0 | 60 (74.1%) | 60 (74.1%) |  |
| 1 | 21 (25.9%) | 21 (25.9%) |  |
| Metastasis status |  |  | 1.000 |
| No | 68 (83.9%) | 68 (83.9%) |  |
| Yes | 13 (16.1%) | 13 (16.1%) |  |
| Degree of differentiation |  |  | 1.000 |
| Well and moderately differentiated | 66 (81.5%) | 66 (81.5%) |  |
| Poorly differentiated | 15 (18.5%) | 15 (18.5%) |  |
| TNM stage |  |  | 1.000 |
| I | 3 (3.7%) | 3 (3.7%) |  |
| II | 32 (39.5%) | 32 (39.5%) |  |
| III | 33 (40.7%) | 33 (40.7%) |  |
| IV | 13 (16.1%) | 13 (16.1%) |  |

Supplementary table 6: Univariate and multivariate analyses of prognostic factors for OS of 162 CRC patients after PSM

| Variable | Univariate | | Multivariate | | | | | | |
| --- | --- | --- | --- | --- | --- | --- | --- | --- | --- |
|  | Log-rank χ^2^ | P value | B | | | SE | HR | 95% CI | *P* value |
| Gender (male/female) | 0.814 | 0.367 | |  |  | |  |  |  |
| Age, years (> 57/≤ 57) | 0.583 | 0.445 | |  |  | |  |  |  |
| Lymph node metastasis (N0-1/N2) | 28.795 | < 0.001* | | 0.462 | 0.366 | | 1.588 | 0.775 to 3.255 | 0.207 |
| Vascular thrombosis (Yes/No) | 34.230 | <0.001* | | 0.356 | 0.413 | | 1.428 | 0.635 to 3.211 | 0.389 |
| Pathology differentiation (Well and moderately differentiated/poor differentiation) | 9.115 | 0.003* | | 0.620 | 0.419 | | 1.858 | 0.818 to 4.233 | 0.139 |
| TNM (AJCC 7th)  (I-II/III-IV) | 20.888 | <0.001* | | 0.489 | 0.441 | | 1.631 | 0.686 to 3.874 | 0.268 |
| Invasion depth (Whole layer/ no whole layer) | 1.529 | 0.216 | | 0.503 | 1.032 | | 1.654 | 0.219 to 12.511 | 0.626 |
| Nerve invasion (yes /no) | 26.784 | <0.001* | | 0.880 | 0.396 | | 2.411 | 1.110 to 5.237 | 0.026 |
| Metastasis status(yes/no) | 24.908 | <0.001* | | 0.653 | 0.456 | | 1.920 | 0.785 to 4.698 | 0.153 |
| EIF4A2(high expression/low expression) | 5.623 | 0.018* | | 0.680 | 0.284 | | 1.973 | 1.130 to 3.443 | 0.017 |

OS: overall survival, CRC: colorectal cancer, PSM: propensity score matching

Supplementary table 7: Gene lists of Metastasis array

| METAP2 | RPL13A | RB1 | PTEN | RPSA | DENR | CD44 | TNFSF10 | GNRH1 | HTATIP2 | HPRT1 | IL18 |
| --- | --- | --- | --- | --- | --- | --- | --- | --- | --- | --- | --- |
| SMAD4 | NME1 | MDM2 | NME2 | SET | KRAS | CCL7 | SMAD2 | IL1B | B2M | ETV4 | CXCR4 |
| CTSK | MTSS1 | PNN | CTSL1 | VEGFA | TIMP4 | MET | MMP7 | TRPM1 | MMP10 | MYCL1 | MMP3 |
| CDKN2A | RORB | HPSE | CXCR2 | APC | SSTR2 | HGF | FXYD5 | CDH11 | KISS1 | MYC | MMP13 |
| MGAT5 | SYK | KISS1R | CDH1 | IGF1 | NR4A3 | CDH6 | CXCL12 | CST7 | TP53 | TIMP2 | MTA1 |
| ITGB3 | ITGA7 | TSHR | FN1 | NME4 | GAPDH | CTNNA1 | TIMP3 | MMP9 | FGFR4 | MMP11 | PLAUR |
| FAT1 | MCAM | TGFB1 | MMP2 | FLT4 | CD82 | EPHB2 | TCF20 | COL4A2 | BRMS1 | SRC | NF2 |
| CHD4 | ACTB | HRAS | EWSR1 | CTBP1 |  |  |  |  |  |  |  |
